# ^18^F-meta-fluorobenzylguanidine (^18^F-mFBG) to monitor changes in norepinephrine transporter expression in response to therapeutic intervention in neuroblastoma models

**DOI:** 10.1038/s41598-020-77788-3

**Published:** 2020-12-01

**Authors:** Stephen Turnock, David R. Turton, Carlos Daniel Martins, Louis Chesler, Thomas C. Wilson, Véronique Gouverneur, Graham Smith, Gabriela Kramer-Marek

**Affiliations:** 1 Preclinical Molecular Imaging, Division of Radiotherapy and Imaging, The Institute of Cancer Research, 123 Old Brompton Road, London, SW7 3RP UK; 2 PET Radiochemistry, Division of Radiotherapy and Imaging, The Institute of Cancer Research, 123 Old Brompton Road, London, SW7 3RP UK; 3 Division of Clinical Studies, The Institute of Cancer Research, 123 Old Brompton Road, London, SW7 3RP UK; 4 Department of Chemistry, University of Oxford, 12 Mansfield Road, Oxford, OX1 3TA UK

**Keywords:** Cancer, Biomarkers, Medical research, Oncology

## Abstract

Targeted radiotherapy with ^131^I-mIBG, a substrate of the human norepinephrine transporter (NET-1), shows promising responses in heavily pre-treated neuroblastoma (NB) patients. Combinatorial approaches that enhance ^131^I-mIBG tumour uptake are of substantial clinical interest but biomarkers of response are needed. Here, we investigate the potential of ^18^F-mFBG, a positron emission tomography (PET) analogue of the ^123^I-mIBG radiotracer, to quantify NET-1 expression levels in mouse models of NB following treatment with AZD2014, a dual mTOR inhibitor. The response to AZD2014 treatment was evaluated in *MYCN* amplified NB cell lines (Kelly and SK-N-BE(2)C) by Western blot (WB) and immunohistochemistry. PET quantification of ^18^F-mFBG uptake post-treatment in vivo was performed, and data correlated with NET-1 protein levels measured ex vivo. Following 72 h AZD2014 treatment, in vitro WB analysis indicated decreased mTOR signalling and enhanced NET-1 expression in both cell lines, and ^18^F-mFBG revealed a concentration-dependent increase in NET-1 function. AZD2014 treatment failed however to inhibit mTOR signalling in vivo and did not significantly modulate intratumoural NET-1 activity. Image analysis of ^18^F-mFBG PET data showed correlation to tumour NET-1 protein expression, while further studies are needed to elucidate whether NET-1 upregulation induced by blocking mTOR might be a useful adjunct to ^131^I-mIBG therapy.

## Introduction

Neuroblastoma (NB) is an early childhood cancer derived from abnormal differentiation of sympathoadrenal neural crest. It is the most common extracranial cancer of young children, with approximately 80–100 new cases in the UK per year^1,2^. At diagnosis, approximately 50% of NBs are classified as high risk (HR-NB) with widespread metastases and most commonly associated with amplification or overexpression of the *MYCN* oncogene^3,4^. These patients undergo intense multimodal therapy; and yet event-free survival (EFS) and overall survival (OS) remain below 50%^5,6^.

*MYCN* amplification in NB is associated with disseminated disease and poor prognosis. Currently there are no *MYCN* targeting agents in routine clinical use, although the NB differentiating agent 13-cis-retinoic acid (isotretinoin; 13-cisRA) has shown *MYCN* disruption in vitro^3,7^. However, other treatments that target *MYCN* transcription, or synthetic lethal interactions with and stabilisation of MYCN protein are being investigated in clinical trials^8,9^. The PI3K/Akt/mTOR axis is of particular interest due to its central role in NB cell growth, proliferation and survival, and in MYCN potentiation^10–13^; and PI3K/mTOR/Akt inhibitors have shown efficacy in MYCN degradation in several NB animal models^9,14,15^. However, clinical trials in children have been limited, potentially owing to dose limiting toxicities and mixed responses in adults^16–18^.

As they are derived from sympathoadrenal precursors, neuroblasts in NB are characterised by expression of surface noradrenaline transporters (NET-1)^19^. The noradrenaline analogue, meta-iodobenzylguanidine, radiolabelled with either iodine-123 (^123^I-mIBG) or iodine-131 (^131^I-mIBG), has been widely used as a theranostic pair for detection of NB and treatment of refractory/recurrent NB, owing to its specific targeting of NET-1^20,21^. Although 90% of NB tumours are mIBG avid, clinical response to ^131^I-mIBG targeted radiotherapy is variable (from 0 to 57%)^22–24^. It has been shown that higher doses of ^131^I-mIBG produce more favourable outcomes in NB patients but at the same time, may cause severe haematological toxicities that limit this approach^22,23^.

Therapies that target NET-1 and increase ^131^I-mIBG uptake into the tumour cells are under investigation. For example, non-carrier-aided (NCA) ^131^I-mIBG increases the radioactive concentration of mIBG entering the target cell^24,25^. Furthermore, anticancer drugs (e.g. vincristine, irinotecan, or vorinostat), in combination with ^131^I-mIBG, may sensitise cells to DNA damage^26,27^ and increase ^131^I-mIBG uptake through enhanced NET-1 expression and function. The mechanisms by which NET-1 expression is regulated in NB are still unclear. However, it has been hypothesised that an increased NET-1 level following vorinostat-targeted actions on HDAC may proceed through disruption of HDAC interactions with protein phosphatase 1, causing subsequent dephosphorylation of Akt at serine 473 (S473)^28^. Furthermore, it has been reported that targeted inhibition of the Akt protein^29^ and deletion of mTORC2 may lead to a marked increase in the NET-1 expression^30^.

Although ^123^I-mIBG is suitable to specifically visualise NET-1 positive lesions, SPECT imaging has lower detection sensitivity than positron emission tomography (PET). Indeed, the PET analogues of mIBG, namely ^18^F-mFBG and ^124^I-mIBG, have shown greater lesion detection compared with ^123^I-mIBG^31–33^. Additionally, PET allows for more accurate radiotracer quantification within the delineated tissue structures. Iodine-124 is a good surrogate for iodine-131 dosimetry owing to their chemical and half-life similarities (4.2 d and 8.02 d for iodine-124 and iodine-131, respectively)^34^. However, iodine-124 has a rather complex decay scheme with a positron branching ratio of only 23% and a high incidence of prompt gammas that interfere with image quantification^35,36^. In contrast, fluorine-18 has a short half-life of 109.7 min and the positron branching ratio of 96.9%, which allows for post-imaging assessment to be undertaken within hours rather than days, thereby improving patient well-being^31^. Taken together, these factors highlight the need for development of F-18-based imaging biomarkers to monitor therapeutic response in NB.

Until recently, the use of ^18^F-mFBG has been limited mostly because of its multi-step synthesis, which initially had to be performed manually^37^. In 2014, Zhang et al. reported an updated radiosynthesis of this agent that required 3 steps and 3 h to end of synthesis. The pure product was achieved with a decay-corrected radiochemical yield (RCY) of roughly 11% and a molar activity of about 18 GBq/µmol^38^. Since then, simplified radiosynthetic approaches have been developed and applied to the production of this radiotracer for clinical use^39–42^.

Herein, we report a 2-step automated synthesis of ^18^F-mFBG. The radiotracer was used to measure NET-1 expression changes in response to therapeutic intervention in NB cells and xenograft models. In particular, we explored whether targeted inhibition of the mTOR/Akt axis could enhance NET-1 expression through downregulation of p-Akt^S473^ using the dual mTOR complex 1 and 2 (mTORC1/2) inhibitor AZD2014 in *MYCN* amplified NB models in vitro and in vivo. Finally, we sought to assess modulation of NET-1 using ^18^F-mFBG image analysis.

## Results

### Internalisation of ^18^F-mFBG and ^123^I-mIBG

**Figure 1:**
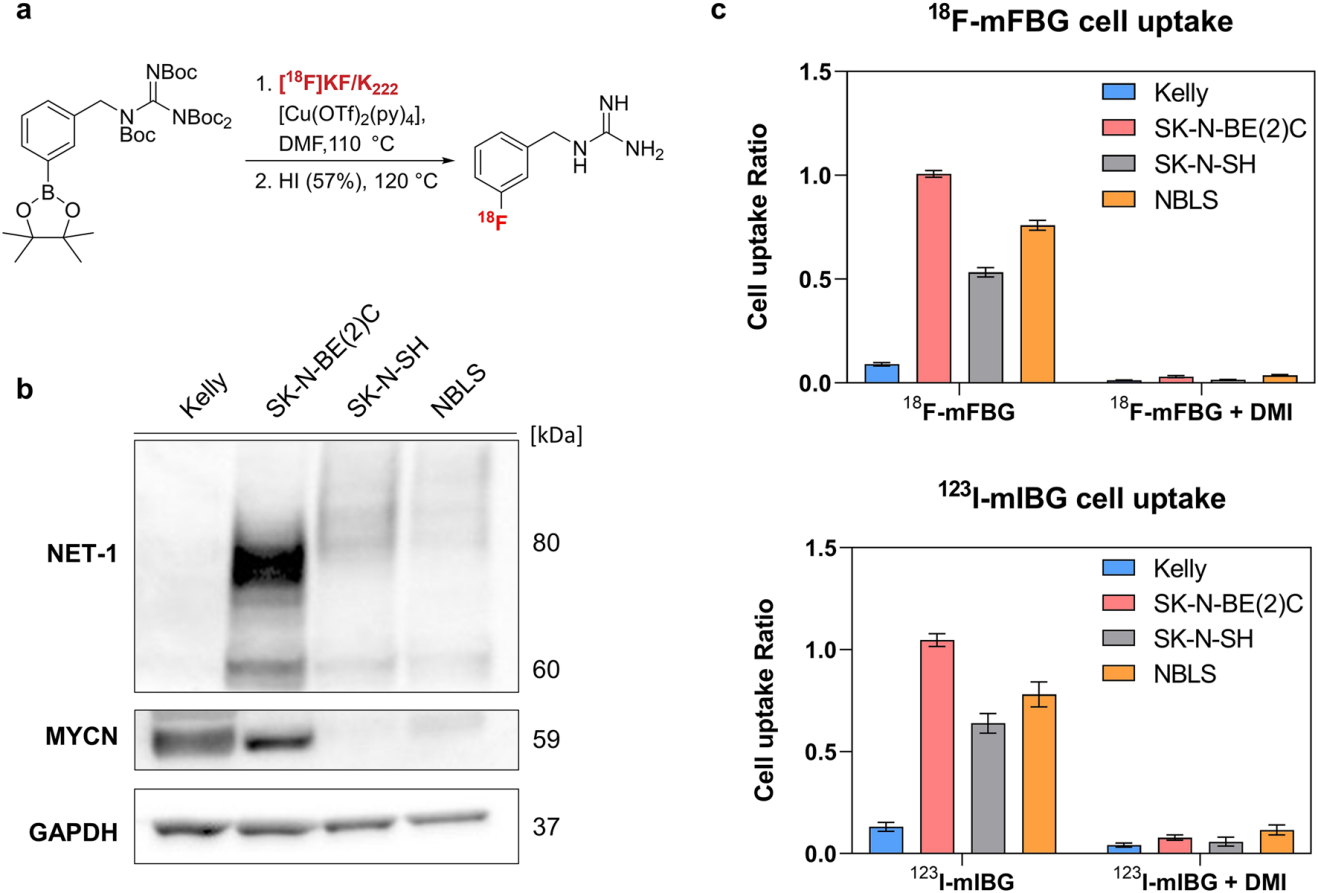
(**a**) Simplified radiosynthesis scheme of ^18^F-mFBG by copper-mediated deborylation. (**b**) Total NET-1 protein expression in selected NB cell lines determined by Western blot. Full length blots are presented in Supplementary Figure WB1. (**c**) ^18^F-mFBG (top bar chart) and ^123^I-mIBG (bottom bar chart) radiotracer uptake in NB cell lines for 1 h at 37 °C, inhibited with and without 50 µM desipramine (DMI; 15 min pre-incubation). Data are presented as mean ± SEM, n ≥ 2 per group, performed in triplicate. Graphs are generated using GraphPad Prism (v 8.4.1), https://www.graphpad.com.

To assess the NET-1-mediated uptake of ^18^F-mFBG (Fig. 1a), levels of NET-1 protein expression were confirmed in a representative panel of NB cell lines, varying in MYCN expression. From WB analysis, the Kelly cell line (*MYCN*-amplified) had an undetectable expression of NET-1 protein, whereas SK-N-SH and NBLS (*MYCN* diploid) and the SK-N-BE(2)C cell line (*MYCN* amplified, TP53 mutant) were characterised by intermediate and high levels of NET-1 protein, respectively (Fig. 1b). When incubated with either ^123^I-mIBG or ^18^F-mFBG, the cell-associated radioactivity reflected NET-1 protein levels with SK-N-BE(2)C cells showing the greatest uptake and Kelly showing the least uptake (Fig. 1c). In contrast, uptake in cells with intermediate NET-1 expression did not reflect the total protein level measured by WB, perhaps due to the lower number of transporters available on the membrane for radiotracer binding. Importantly, the uptake of ^18^F-mFBG paralleled that of ^123^I-mIBG and the uptake of each product was inhibited by desipramine (DMI), a NET-1 inhibitor, confirming the requirement for NET-1 activity in cellular tracer uptake (Fig. 1c).

### In vivo radiotracer distribution

**Figure 2:**
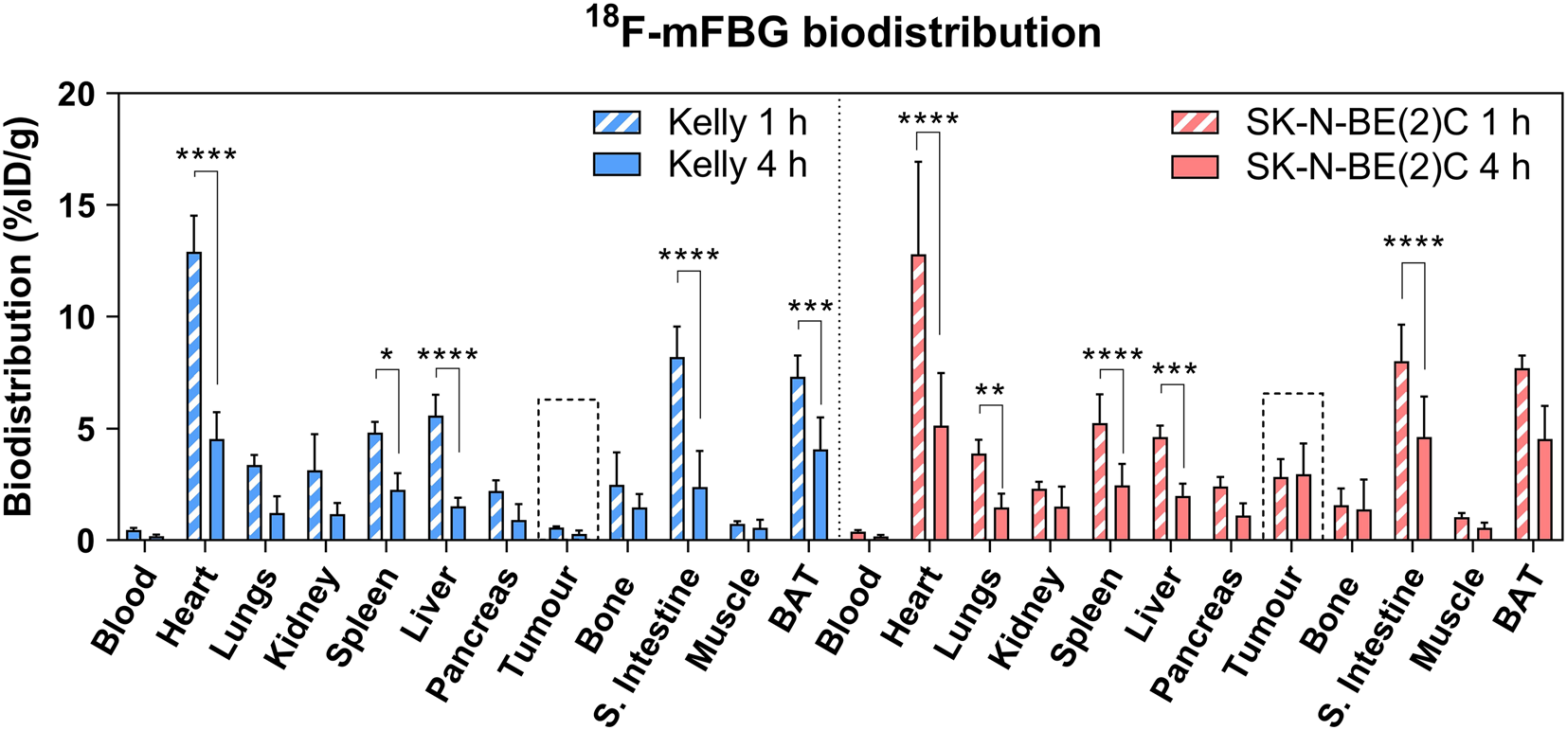
Biodistribution of ^18^F-mFBG 1 and 4 h after injection of mice bearing SK-N-BE(2)C or Kelly xenografts. Data are presented as mean ± SD, n ≥ 3 per group. *p < 0.05, **p < 0.01, ***p < 0.001, ****p < 0.0001; 2-way ANOVA with Tukey post-hoc test. S. Intestine = Small intestine, BAT = Brown adipose tissue. Graph is generated using GraphPad Prism (v 8.4.1), https://www.graphpad.com.

Following characterisation in vitro, ^18^F-mFBG distribution was assessed in vivo using Kelly (NET-1 low) and SK-N-BE(2)C (NET-1 high) xenografts. ^18^F-mFBG biodistribution studies showed that radiotracer uptake was particularly high in sympathetically innervated tissues including the heart (> 12.0%ID/g), brown adipose tissue (BAT) (> 7.0%ID/g) and the small intestine (~ 8.0%ID/g) 1 h p.i. (Fig. 2 and Supplementary Table 1). However, significant clearance of radioactivity from these organs was observed at 4 h p.i. in both xenograft models (Fig. 2) (*p* < 0.01, n ≥ 3 per group). In accordance with the higher expression of NET-1, SK-N-BE(2)C xenografts exhibited significantly higher tumour uptake than Kelly xenografts at 4 h p.i. (2.96 ± 1.38%ID/g (n = 5) vs. 0.30 ± 0.14%ID/g (n = 3); *p* = 0.03). Tumour-to-blood and tumour-to-muscle ratios of 17.69 ± 10.00 and 6.24 ± 3.64, respectively, were observed 4 h p.i. in SK-N-BE(2)C xenografts (Supplementary Table 1). When ^123^I-mIBG was investigated in vivo, biodistribution studies (24 h p.i.) revealed similar radiotracer pharmacokinetics, with greatest accumulation of radioactivity in tumour tissue and sympathetically innervated heart, small intestine and BAT at this time point (Supplementary Table 1 and Supplementary Fig. 3). Tumour-to-background ratios were greater for ^123^I-mIBG than ^18^F-mFBG most probably as a consequence of the biodistribution studies being performed at a later time point and likely clearing of the radioactive agent from non-target organs (Supplementary Table 1).

**Figure 3:**
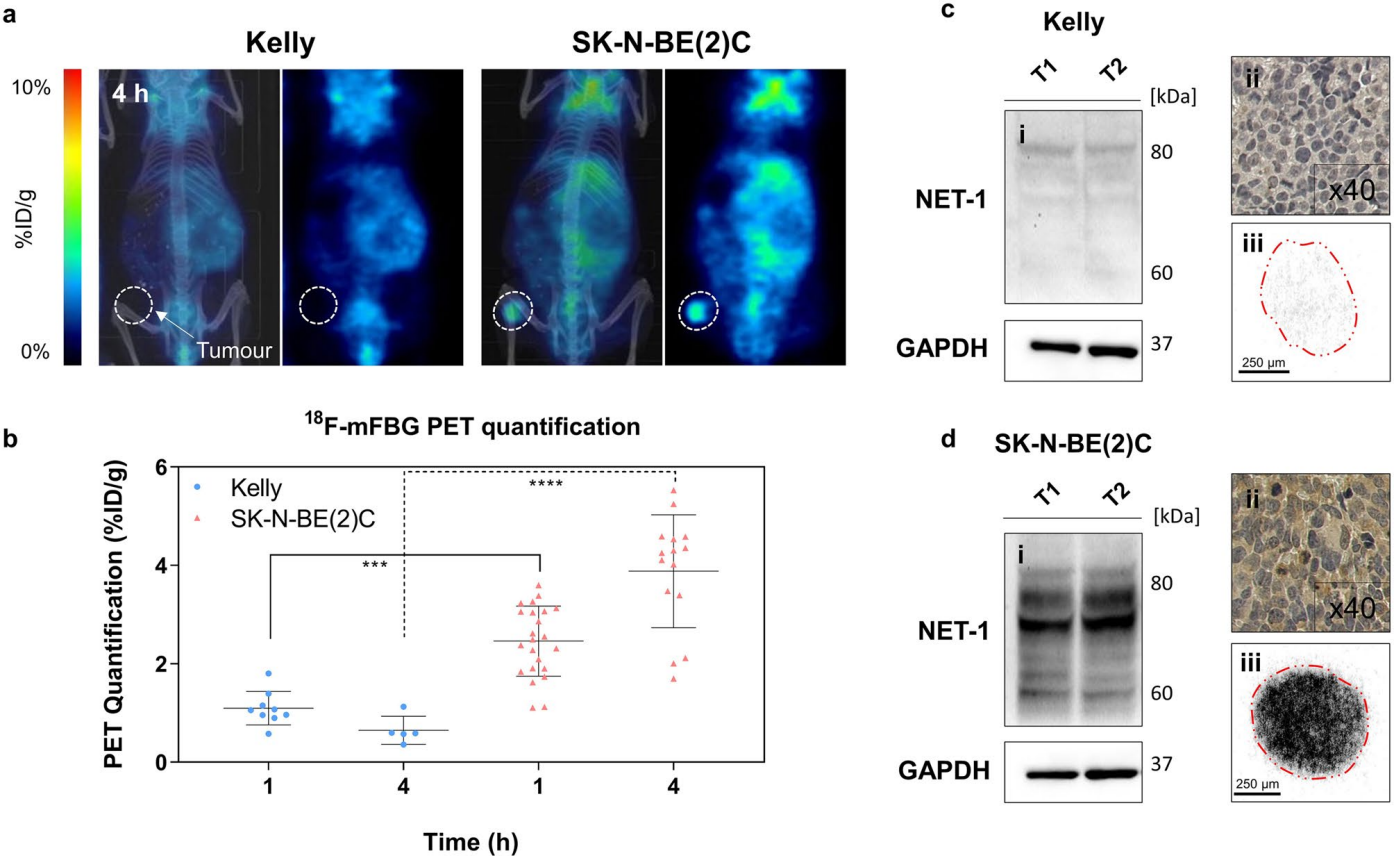
PET analysis of ^18^F-mFBG distribution and NET-1 expression profile. (**a**) Representative coronal slice of Kelly (left) and SK-N-BE(2)C (right) xenograft PET images with corresponding maximum intensity projection CT overlay, 4 h p.i. of ^18^F-mFBG. Dotted circles represent tumour region. (**b**) PET quantification of Kelly and SK-N-BE(2)C xenografts 1 and 4 h p.i. of ^18^F-mFBG. Data are presented as mean ± SD, n ≥ 5 per group, each dot represents one tumour. ***p < 0.001, ****p < 0.0001; Student’s t-test. Ex vivo analysis of Kelly (**c**) and SK-N-BE(2)C (**d**) xenografts (i) Western blot (n = 2) (Full-length blots are presented in Supplementary Figure WB2) (ii) immunohistochemistry of NET-1 protein (× 40) and (iii) autoradiography exposition of tumour section, 2 h on to X-ray film. Graph is generated using GraphPad Prism (v 8.4.1), https://www.graphpad.com.

PET/CT scans performed 1 and 4 h p.i. of ^18^F-mFBG clearly delineated tumour masses in SK-N-BE(2)C tumour bearing mice (Fig. 3a). After quantitative analysis of PET images, SK-N-BE(2)C tumours showed higher (*p* < 0.001) tumour uptake (2.46 ± 0.71%ID/g (n = 21) and 3.88 ± 1.15%ID/g (n = 15) at 1 and 4 h p.i., respectively) than in Kelly xenografts (1.10 ± 0.34 (n = 9) and 0.65 ± 0.29%ID/g (n = 5) at 1 and 4 h p.i., respectively), (Fig. 3b). Quantitative PET analysis of tumour uptake correlated to ex vivo measurements of radiotracer accumulation using gamma counter (linear regression (r^2^ = 0.72), (Supplementary Fig. 4a). Additionally, both WB and IHC confirmed low expression of NET-1 in Kelly xenografts and high NET-1 expression in the SK-N-BE(2)C tumours (Fig. 3c,d). This paralleled target expression levels defined in vitro. Autoradiography images of SK-N-BE(2)C tumour slices showed a high and uniform distribution of ^18^F-mFBG. Conversely, the radiotracer signal in the Kelly tumour sections was low confirming the association between tumour ^18^F-mFBG uptake and NET-1 protein expression (Fig. 3c,d).

### In vitro mTOR inhibition and modulation of radiotracer uptake

**Figure 4:**
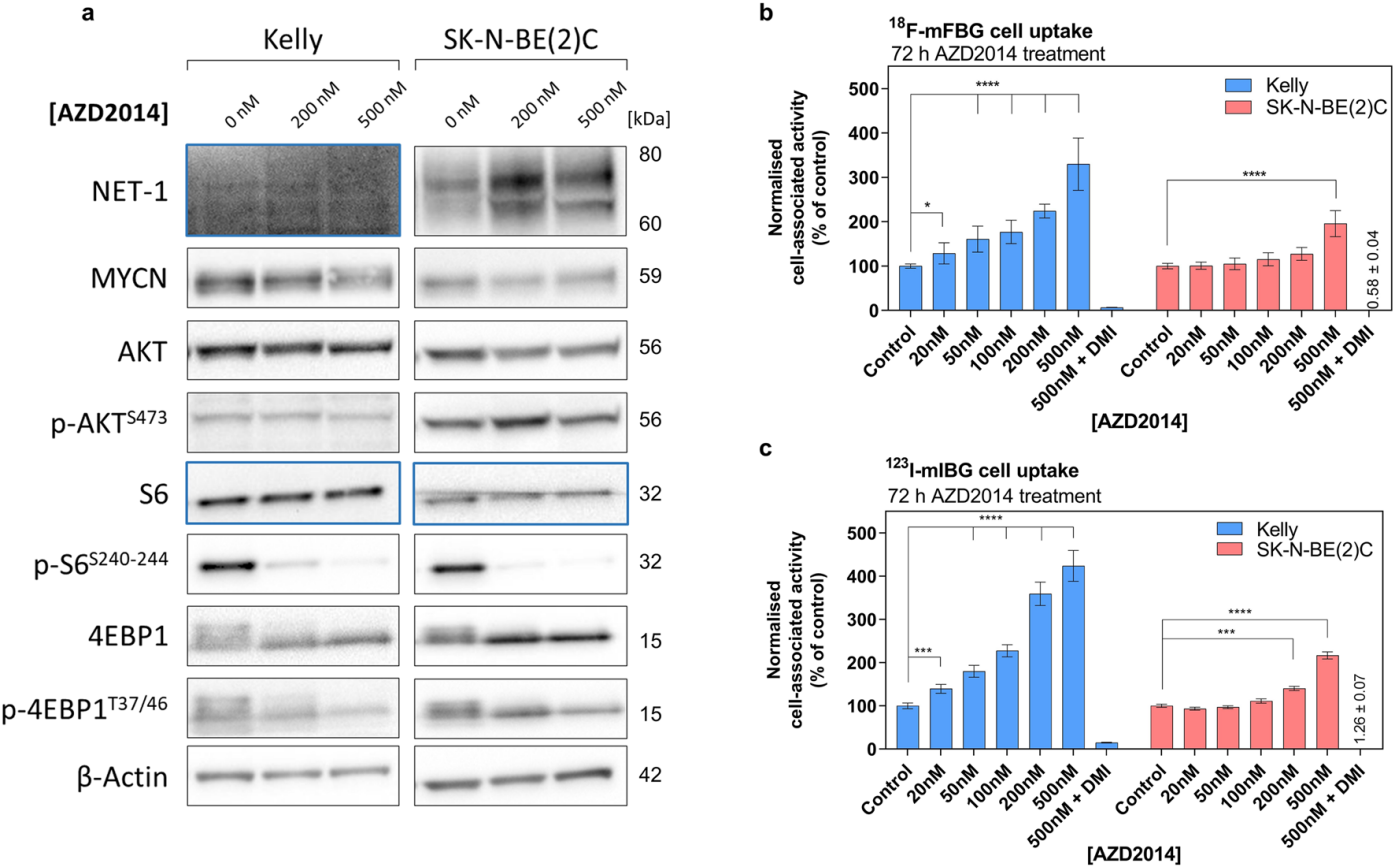
(**a**) Western blot analysis of key protein expression in Kelly and SK-N-BE(2)C cells after incubation with 0–500 nM AZD2014 for 72 h. Blue boxes indicate a second membrane for better protein signal acquisition. Full-length blots are presented in Supplementary Figure WB5 and WB6. (**b**) ^18^F-mFBG uptake in Kelly and SK-N-BE(2)C cells pre-treated with AZD2014 for 72 h 0–500 nM, and the same conditions for (**c**) ^123^I-mIBG uptake. Data presented as mean ± SEM, n ≥ 2, performed in triplicate. Inhibition of radiotracer uptake n = 1, performed in triplicate. *p < 0.05, ***p < 0.001, ****p < 0.0001; 2-way ANOVA with Tukey post-hoc test. DMI = desipramine. Graphs are generated using GraphPad Prism (v 8.4.1), https://www.graphpad.com.

To assess the cellular response to mTORC1/2 inhibition, Kelly and SK-N-BE(2)C cell lines, both of which over-express MYCN protein (Fig. 1b), were incubated with AZD2014 (0 to 1 µM) for 72 h. The GI_50_ for AZD2014 (concentration that reduced cell viability by 50%) was 476 nM in Kelly and 307 nM in SK-N-BE(2)C cells (Supplementary Fig. 5). Key substrates of AZD2014- induced mTORC1/2 inhibition were assessed by WB. The immunoblots showed a concentration dependent decrease in phosphorylation of both ribosomal S6 protein (S6) and eukaryotic translation initiation factor 4E-binding protein 1 (4EBP1) in Kelly and SK-N-BE(2)C cells (Fig. 4a, Supplementary Fig. 6 and 7), key indicators of mTORC1 inhibition. MYCN protein expression was not robustly reduced (Fig. 4a, Supplementary Fig. 6 and 7). In both cell lines, a concentration-dependent downregulation of p-Akt^S473^ was evident at early time points (3–12 h) (Supplementary Fig. 6 and 7), but inhibition was not sustained after 72 h incubation with the compound (Fig. 4a).

Given the potential role of Akt in NET-1 protein synthesis and surface expression^43^, we further looked at NET-1 protein levels following AZD2014 treatment. In SK-N-BE(2)C cells, a prominent and persistent increase in total NET-1 protein expression was seen at 24 h post-incubation (Supplementary Fig. 6) and was maintained at 72 h (Fig. 4a). In Kelly cells, the WB analysis was not sensitive enough to detect and accurately quantify the inherently low expression of the target protein.

To investigate potential changes in NET-1 function following an AZD2014-induced increase in protein expression, ^18^F-mFBG and ^123^I-mIBG cell uptake assays were performed in Kelly and SK-N-BE(2)C cells incubated with the drug (0–500 nM) for 24 and 72 h. A concentration-dependent increase of ^123^I-mIBG and ^18^F-mFBG uptake was observed in both cell lines in the 24 h treatment-time point (Supplementary Fig. 8b), but this was more evident at 72 h as compared to untreated control cells (Fig. 4b,c). Following pre-treatment with 200 nM and 500 nM AZD2014, uptake of ^18^F-mFBG in Kelly cells significantly increased by 128 ± 23% and 292 ± 27%, respectively (*p* < 0.0001, n = 2 performed in triplicate). Perhaps due to the already high expression of NET-1, increase in radiotracer uptake was less evident in SK-N-BE(2)C cells (39 ± 9% and 62 ± 5%, respectively). A similar trend was seen in ^123^I-mIBG incubated cells pre-treated with AZD2014 (Fig. 4c). Desipramine (500 nM) reduced the radioactivity signal in AZD2014 pre-treated cells confirming that uptake of both radiotracers was NET-1 transporter-specific (Fig. 4b,c Supplementary Fig. 8a).

### ^18^F-mFBG to monitor changes in NET-1 expression in vivo

In light of the effects of AZD2014 treatment on NET-1 expression observed in vitro, we investigated whether ^18^F-mFBG could capture changes in tumour NET-1 expression following AZD2014 (25 mg/kg/day) treatment in Kelly xenografts (low NET-1). After 3 days post-treatment initiation mild toxicities were observed (i.e. mice treated with the compound lost ~ 10% of body weight compared to the vehicle control) (Supplementary Fig. 9a). Moreover, tumour growth was impeded in AZD2014-treated xenografts (Supplementary Fig. 9c) (n = 3, *p* < 0.0001). Biodistribution studies performed 4 h post-radiotracer injection demonstrated no change in radiotracer uptake in organs such as the heart or small intestine, which are known to be innervated by NET-1-positive sympathetic ganglia^44^. Further, we observed an increase in ^18^F-mFBG uptake in drug treated tumours (0.74 ± 0.25%ID/g) as compared to controls (0.39 ± 0.13), but this difference was not significant (*p* = 0.85, n = 3). The tumour-to-blood and tumour-to-muscle ratios were 1.75 ± 0.29 and 0.53 ± 0.01 in the vehicle group, as compared with 4.16 ± 1.89 and 1.23 ± 0.61 in the AZD2104 treated mice, respectively (Table 1).

**Table 1:** Biodistribution of ^18^F-mFBG 4 h after injection of mice bearing Kelly xenografts, pre-treated with AZD2014 (25 mg/kg/day) or vehicle control (DMSO 10%, PEG400 90%) for 3 days.

Kelly	^18^F-mFBG biodistribution 4 h p.i. (mean %ID/g ± SD)	
	Vehicle	AZD2014 (25 mg/kg/day)
*N* =	*3*	*3*
Blood	0.22 ± 0.05	0.19 ± 0.03
Heart	6.34 ± 1.41	4.22 ± 0.85
Lungs	1.52 ± 0.22	1.31 ± 0.39
Kidney	1.11 ± 0.47	0.97 ± 0.17
Spleen	3.77 ± 0.82	4.58 ± 0.77
Liver	2.37 ± 0.71	2.08 ± 0.45
Pancreas	1.26 ± 0.28	1.25 ± 0.21
Tumour	0.39 ± 0.13	0.74 ± 0.25
Bone	1.43 ± 0.83	0.99 ± 0.07
Small intestine	6.57 ± 1.12	7.30 ± 1.11
Muscle	0.74 ± 0.23	0.64 ± 0.17
Brown adipose	5.84 ± 2.44	7.20 ± 0.88
Tumour:blood	1.75 ± 0.29	4.16 ± 1.89
Tumour:muscle	0.53 ± 0.01	1.23 ± 0.61

Data are presented as mean ± SD, n = 3 per group.

**Figure 5:**
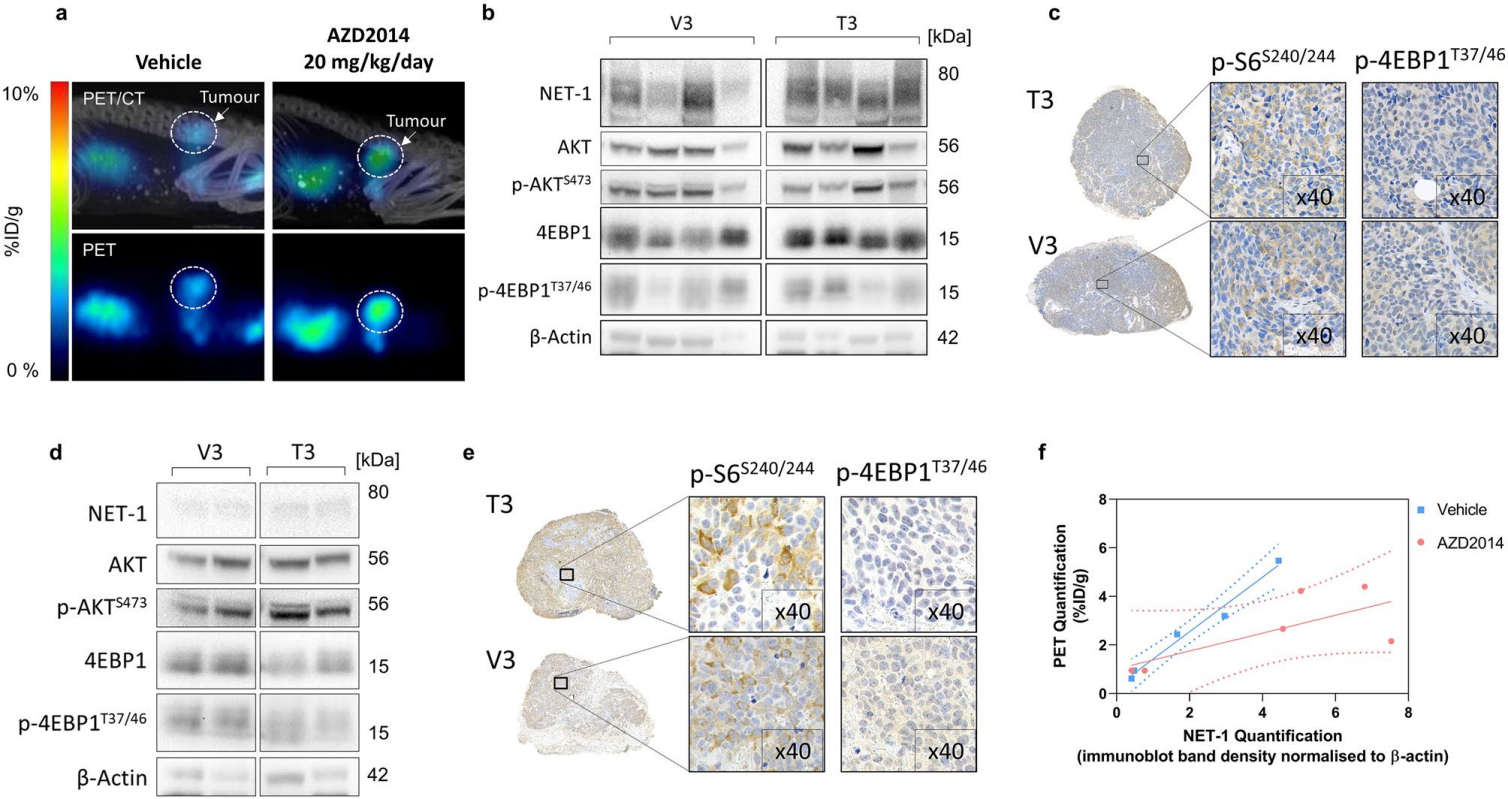
In vivo assessment of AZD2014 and ^18^F-mFBG uptake. (**a**) PET/CT of SK-N-BE(2)C xenografts. PET image and corresponding maximum intensity projection CT overlay, 4 h p.i. of ^18^F-mFBG in vehicle (left) and AZD2014 (20 mg/kg/day)(right) treated mice, 3 days. Tumours highlighted with dotted white circle. (**b**) Western blot (n = 4) and (**c**) IHC analysis of SK-N-BE(2)C tumour lysates of animals treated for 3 days with vehicle (V3) or AZD2014 20 mg/kg/day (T3). (**d**) Western blot (n = 2) and (**e**) IHC analysis of Kelly tumour lysates treated for 3 days with vehicle (V3) or AZD2014 25 mg/kg/day (T3). (**f**) Linear regression fit of NET-1 band density vs PET mean quantification in vehicle (r^2^ = 0.98) and AZD2014 (r^2^ = 0.53) treated tumours (n = 5–6 per group). For Western blots, full-length blots are presented in Supplementary Figures WB3 and WB4. Graph is generated using GraphPad Prism (v 8.4.1), https://www.graphpad.com.

Following these observations, we evaluated a lower dose (20 mg/kg/day) of AZD2014 (or vehicle control) for 1, 3 or 7 days in SK-N-BE(2)C tumour bearing mice to ascertain longitudinal tumour NET-1 status. No toxicity was observed during the treatment period. No change in non-target organ distribution was observed at any time-point (Table 2). A prominent difference in tumour radiotracer accumulation was observed on day 3 in the treatment group (3.95 ± 1.53%ID/g) as compared to the vehicle controls (2.39 ± 0.01%ID/g) resulting in a greater tumour-to-blood and tumour-to-muscle ratio (Table 2), however this was statistically insignificant (*p* = 0.81 and *p* > 0.99, respectively, n = 3). These results were concordant with image-derived tumour radiotracer uptake studies (Fig. 5a, Supplementary Fig. 4b). We observed a decrease in radioactivity in treated tumours on day 7 post-treatment (2.61 ± 1.19%ID/g) that was statistically indistinguishable from controls (2.17 ± 1.01%ID/g) (*p* > 0.99, n = 3). These statistically insignificant changes in radiotracer uptake between vehicle and AZD2014-treated xenografts were corroborated by ex vivo analysis of tumour samples. Western blot data showed an increase in NET-1 expression in SK-N-BE(2)C tumour lysates (Fig. 5b), but this difference was not significant (*p* = 0.07, n = 4) (Supplementary Fig. 10). Moreover, there were no apparent differences in phosphorylated Akt between control and treated tumours of both tumour types. Furthermore, staining of phosphorylated S6 or 4EBP1 was identical in SK-N-BE(2)C tumours treated with AZD2014 and vehicle (Fig. 5c). In Kelly xenografts, there was no difference between NET-1 expression levels in Kelly tumour lysates between control and treated animals (Fig. 5d) (*p* = 0.84, n = 2). Further, IHC showed no changes in p-S6^S240/244^ or p-4EBP1^T37/46^ in Kelly xenografts (Fig. 5e) indicating that the dose of AZD2014 in this regimen failed to inhibit mTORC1/2. Quantitative PET analysis did demonstrate good correlation with tumour NET-1 status in the vehicle treated tumours (r^2^ = 0.98), but in the AZD2014 treated animals, the slope of the line of best fit was perturbed (r^2^ = 0.53) (Fig. 5f).

**Table 2:** Biodistribution of ^18^F-mFBG 4 h after injection of mice bearing SK-N-BE(2)C xenografts, pre-treated with AZD2014 (20 mg/kg/day) or vehicle control (DMSO 10%, PEG400 90%) for 1, 3 or 7 days.

SK-N-BE(2)C	^18^F-mFBG biodistribution 4 h p.i. (%ID/g)					
	1 day vehicle	1 day AZD2014 (20 mg/kg/day)	3 day vehicle	3 Day AZD2014 (20 mg/kg/day)	7 day vehicle	7 day AZD2014 (20 mg/kg/day)
*N* =	*3*	*3*	*3*	*3*	*3*	*3*
Blood	0.18 ± 0.02	0.18 ± 0.04	0.18 ± 0.08	0.16 ± 0.02	0.17 ± 0.06	0.32 ± 0.14
Heart	4.38 ± 1.68	4.34 ± 2.38	3.64 ± 0.89	3.20 ± 0.43^a^	5.87 ± 2.80	4.17 ± 1.63
Lungs	1.22 ± 0.37	1.27 ± 0.68	1.15 ± 0.23	1.04 ± 0.12	1.86 ± 0.64	1.33 ± 0.36
Kidney	0.79 ± 0.36	0.98 ± 0.04	1.13 ± 0.47	0.89 ± 0.13	1.09 ± 0.16	1.02 ± 0.15
Spleen	3.19 ± 0.63	2.93 ± 0.82	2.08 ± 1.29	2.27 ± 1.06	1.75 ± 0.67	1.64 ± 0.46
Liver	1.94 ± 0.30	1.63 ± 0.69	1.96 ± 0.02	1.74 ± 0.23	2.15 ± 0.30	2.64 ± 0.46
Pancreas	1.01 ± 0.18	1.09 ± 0.42	1.25 ± 0.51	1.29 ± 0.51	2.81 ± 1.72	3.08 ± 1.80
Tumour	2.76 ± 1.14	3.29 ± 1.01	2.39 ± 0.10	3.95 ± 1.53	2.17 ± 1.01	2.61 ± 1.19
Small intestine	4.50 ± 0.24^b^	4.79 ± 1.21	5.32 ± 0.11	4.88 ± 1.28	6.22 ± 0.35	6.87 ± 1.86
Muscle	0.60 ± 0.13	0.59 ± 0.16	0.53 ± 0.19	0.55 ± 0.09	0.67 ± 0.07	0.75 ± 0.02
Brown adipose	5.14 ± 0.38^b^	5.47 ± 0.45^b^	5.15 ± 0.78^b^	5.32 ± 1.51^b^	6.08 ± 2.45	8.08 ± 0.72
Bone	4.98 ± 2.18^c^	4.55 ± 2.15^c^	1.64 ± 0.45	2.42 ± 2.57	1.13 ± 0.20	0.63 ± 0.54
Tumour:blood	15.27 ± 5.32	17.72 ± 2.35	14.47 ± 4.61	23.76 ± 6.20	15.55 ± 13.55	8.06 ± 2.57
Tumour:muscle	4.44 ± 0.94	5.74 ± 1.77	4.89 ± 1.48	7.06 ± 1.79	3.36 ± 1.90	3.50 ± 1.62

Data are presented as mean ± SD, n = 3 per group.

a = *p* < 0.05 compared to 7-day vehicle group of the same tissue.

b = *p* < 0.05 compared to 7-day AZD2014 group of the same tissue.

c = *p* < 0.05 compared to 3-day and 7-day groups of the same tissue.

## Discussion

Therapies for patients with HR-NB which aim to block the activity of the *MYCN* through inhibition of the mTOR/Akt axis are of clinical interest^8,45,46^. This pathway is aberrantly expressed in many cancers including NB^12,13,47^, however, mTOR/Akt inhibitors have yet to provide robust tumour control^48,49^. Interestingly, Dubois et al*.* reported that patients with *MYCN* amplification have comparatively lower NET-1 protein levels than those who are *MYCN* non-amplified^50^, suggesting that *MYCN* amplification impedes mature neural cell features such as NET-1 expression^51^. Therefore, a combined strategy by which *MYCN* is inhibited and NET-1 levels could be primed may be of particular benefit.

The radioconjugates ^123^I- and ^131^I-mIBG are widely used for the imaging and therapy of NB patients owing to the selective presence of NET-1, and a ^123^I-mIBG baseline scan is a necessary prerequisite of ^131^I-mIBG therapy. ^123^I-mIBG is also used to monitor disease progression, and increased accumulation of the radiotracer is associated with an unfavourable outcome^52^. Furthermore, an early response to chemotherapy captured on mIBG imaging correlates with good prognosis, improved EFS and OS in advanced and HR-NB^53^. However, ^123^I-mIBG imaging is a descriptive technique, making clinical interpretation and accurate quantitation of a the dose:response relationship challenging. Semi-quantitative scoring systems have been developed (e.g. CURIE or SIOPEN visual scoring system) to provide an objective and uniform way for evaluation of disease burden and efficacy of therapies^54–56^. The availability of quantitative PET imaging agents to measure NET-1 activity would improve detection and quantitation of the disease.

Promising data in 5 NB patients indicates that a fluorinated guanidine analogue, ^18^F-mFBG, has similar biodistribution to that of ^123^I-mIBG, faster clearance, higher imaging resolution, and improved assessment of lesion radiotracer uptake^31^. Of note, a new European clinical trial is presently investigating ^18^F-mFBG imaging in NET-1 expressing tumours [NCT02348749]. However, these trials have yet to introduce quantifiable measures of ^18^F-mFBG uptake in specific tumour lesions. The studies presented herein strengthen the case that ^18^F-mFBG imaging could be a useful modality for non-invasive assessment of NET-1 status in NB during therapeutic intervention. We have implemented simplified radio-synthetic approaches that have been developed to produce ^18^F-mFBG via fluorination of electron-rich aromatics using copper-mediated fluorodeboronation^42^.

Traditional radiochemical strategies have focused on electron-poor aromatics to carry out nucleophilic aromatic substitution reactions (e.g. Balz-Schieman reaction). However, new radiosynthetic strategies have become recently available. For example, Rotstein et al.reported the synthesis of ^18^F-mFBG via a spirocyclic iodonium(III) ylide (~ 14% RCY)^39^. The groups of Sanford and Gouverneur published approaches to the fluorination of electron-rich aromatics via copper-mediated destannylation or deborylation^42,57^. This has proved a versatile method to access highly functionalised electron-rich PET radiotracers including ^18^F-mFBG^58^. These highly promising results prompted us to explore the fully automated cassette-based radiosynthesis of ^18^F-mFBG to support an intensive programme of research at our institution.

As expected, and in line with previously reported studies^38^, uptake of both ^18^F-mFBG and ^123^I-mIBG correlated with the NET-1 expression levels in vitro and allowed for clear delineation of NET-1 expressing SK-N-BE(2)C xenografts after 4 h p.i..

Using two *MYCN* amplified cell lines (Kelly and SK-N-BE(2)C), we initially focused on assessing the ability of the mTORC1/2 inhibitor AZD2014 to target the mTOR/Akt axis in vitro and subsequently investigated whether mTORC1/2 inhibition will modulate NET-1 expression. In vitro*,* we observed the concentration-dependent inhibition of mTORC1 substrates p-4EBP1^T37/46^ and p-S6^S240/244^. In addition, consistent with previously described results, p-Akt^S473^ was also depressed^59,60^*,* however this was only a transient effect in our experiments. Dual inhibition of both mTOR complexes is associated with MYCN protein degradation^9^, which was mild in Kelly and SK-N-BE(2)C cells treated with 500 nM AZD2014. Although higher drug concentrations may result in further MYCN depression, this would result in greater cell death and impede further analysis.

Importantly, WB analysis revealed a concomitant increase in NET-1 expression in SK-N-BE(2)C cells after AZD2014 treatment; an effect attributable to the drug inhibition of p-Akt^S473^^43^. Of note, an enhanced NET-1 function was also highlighted in both Kelly and SK-N-BE(2)C by the increased cell uptake of ^123^I-mIBG and ^18^F-mFBG, in a concentration-dependent manner. The maximum change in NET-1 activity was observed in Kelly cells and was not clearly evident in SK-N-BE(2)C most likely due to high levels of inherent NET-1 expression.

Changes in NET-1 expression level post-mTOR inhibition have not been investigated yet by molecular imaging. Recently, T1-weighted MRI has been used to detect apoptotic responses 24 h following AZD2014 treatment in NB tumours^61^. Therefore, we assessed whether PET imaging could be used to monitor AZD2014-induced alterations in ^18^F-mFBG tumour uptake by upregulating NB NET-1 expression levels in vivo. Following our in vitro findings, Kelly xenografts were used in vivo as a model system. Inhibitor dose regimens were selected based on previous reports^61,62^. After 3 days treatment, tumour growth plateaued and ^18^F-mFBG biodistribution studies showed increased tumour-to-blood and tumour-to-muscle ratios in AZD2014-treated animals compared to the vehicle-treated controls, however PET signals were still too low for robust quantification and mild toxicities were noted. Therefore, a lower AZD2014 dose was investigated in the SK-N-BE(2)C model. There was a large variation in the treated SK-N-BE(2)C response according to PET and these results were corroborated by ex vivo analysis of tumour tissue. In both Kelly and SK-N-BE(2)C tumour lysates, WB results demonstrated only small differences in NET-1 protein levels and there was no robust inhibition of p-Akt^S473^ in treated tumours. Similarly, IHC staining did not detect any reduction of p-S6^S240/244^ and p-4EBP1^T37/46^, which confirmed that the selected dose regimens did not fully inhibit the mTOR signalling.

Interestingly, a clear relationship between ^18^F-mFBG uptake and NET-1 expression was observed in the vehicle treated tumours. However, in the AZD2014 treated group, accumulation of the radiotracer within the tumour was inhibited. This could suggest that the mTORC1/2 inhibitor was affecting the delivery of the radiotracer, which could be due to anti-angiogenic effects of mTOR inhibition^63^. Further optimisation of this approach, including B.I.D. dosing of AZD2014 or using alternative mTORC1/2 inhibitors, is needed to address the potential benefits of mTORC1/2 inhibition and ^131^I-mIBG combinations.

## Conclusion

In this work, we have shown that dual mTORC1/2 inhibitor (AZD2014) potentiates NET-1 expression in NB cells in vitro. Although the AZD2014 exhibited suboptimal activity in vivo and more studies are required to validate a more effective primary therapeutic strategy to inhibit tumour progression whilst sensitising cells to ^131^I-mIBG therapy, the ability of ^18^F-mFBG to quantify the NET-1 expression was highlighted. This work supports the potential of ^18^F-mFBG to use this tracer in future studies for image-guided therapeutic strategies leading to more robust and durable responses to ^131^I-mIBG radiotherapy.

## Materials and methods

### Cell lines

SK-N-SH, SK-N-BE(2)C, Kelly, NBLS human NB cell lines were confirmed of authenticity by short tandem repeat (STR) DNA profiling analysis (Eurofins Medigenomix, Ebersberg, Germany) and routinely found to be mycoplasma negative (PCR detection kit, Surrey Diagnostics Ltd, Cranleigh, UK). SK-N-SH and SK-N-BE(2)C cell lines were cultured in DMEM (Gibco, Life Technologies, Paisley, UK), and NBLS and Kelly in RPMI1640 (Gibco, Life Technologies, Paisley, UK), supplemented with 10% heat-inactivated foetal bovine serum (FBS, Gibco, Life Technologies, Paisley, UK) and grown as a monolayer at 37 °C in a humidified atmosphere containing 5% CO_2_.

### Western blot

Western blotting (WB) was performed as previously described^64^. Antibodies were obtained from Cell Signalling Technologies (London, UK) unless stated otherwise: GAPDH, beta-actin, p-Akt^S473^, Akt, p-4EBP1^T37/46^, p-S6^S240/244^, S6, N-MYC (Merck Millipore, Watford, UK), NET-1 (mAbTechnologies, Stone Mountain, Georgia), anti-rabbit HRP, anti-mouse HRP. Blots were visualised with SuperSignal West Pico and Femto Chemiluminescent Substrate (Thermo Fisher Scientific, Loughborough, UK) and ChemiDoc XRS + System (BioRad, Watford, UK). Data were processed and band density analysed with ImageJ and Image Lab 6.0 (BioRad, Watford, UK).

### Preparation of radiotracers

^123^I-mIBG was purchased from GE Healthcare (AdreView Amersham, UK). ^18^F-mFBG was prepared using a Trasis All in One (AiO) synthesiser (Trasis SA, Liege, Belgium) housed in a shielded hot cell. A simplified synthesis is detailed in Fig. 1a. Detailed synthesis methodology is included in the Supplementary Material (Supplementary Figs. 1/2).

### In vitro uptake of ^123^I-mIBG and ^18^F-mFBG

To evaluate the radioactive agent uptake specificity, approximately 3.0 × 10^5^ adherent cells were incubated with either ^18^F-mFBG (150 kBq) or ^123^I-mIBG (5 kBq) for 1 h at 37 °C, in the presence or absence of the NET-1 specific inhibitor, desipramine (DMI, 50 µM, Sigma Aldrich, Gillingham, UK). Subsequently, the cells were washed with PBS, trypsinised and the radioactivity was measured using a γ-counter (2480 WIZARD^2^, PerkinElmer, Beaconsfield, UK). For each cell line the cell-associated radioactivity was normalised to the number of cells and then each group was presented as a percentage of the signal acquired for SK-N-BE(2)C cells (mean of n = 3 independent experiments performed in triplicate ± SEM).

To test the response of cell uptake using the mTOR1/2 inhibitor AZD2014 (0–500 nM) (vistusertib, Stratech Scientific, Ely, UK), a stock solution of the drug (10 mM) in dimethyl sulfoxide (DMSO, Sigma Aldrich, Gillingham, UK) was initially prepared and then diluted in culture medium to a final DMSO concentration of < 0.1%. Radiotracer uptake was assessed in cells 24 and 72 h after incubation with AZD2014. The uptake was normalised to cell viability as performed using the CellTiter-Glo assay (Promega, Southampton, UK). The data are expressed as the average of n = 3 independent experiments (performed in triplicate) ± SEM.

### In vivo studies

All studies were conducted in compliance with licenses issued under the UK Animals (Scientific Procedures) Act 1986 and approved by the Institute of Cancer Research Animal Welfare and Ethical Review Body (AWERB) according to the United Kingdom National Cancer Research Institute Guidelines for Animal Welfare in Cancer Research (project license PCC916B22, Animals in Science Regulation Unit, Home Office Science, London, UK)^65^. Female Nu(NCr)-Foxn1nu mice (5–7 weeks; 20–22 g) obtained from an in-house breeding colony were used in the studies. Animals were housed in a temperature-controlled facility with 12 h on–12 h off light cycle and provided water and laboratory rodent food ad libitum. Subcutaneous xenografts were generated by injection of either Kelly (5 × 10^6^/0.1 mL PBS/Matrigel 30% v/v; BD Matrigel Matrix, BD Bioscience, San Jose, California, US) or SK-N-BE(2)C (1 × 10^6^/0.1 mL PBS/Matrigel) into the lower flank. For the treatment studies, AZD2014 stock solution in DMSO was diluted in PEG400 to a final concentration of 2 mg/mL (10% DMSO maximum). When tumours reached ~ 100 mm^3^ (calliper measurements using formula: Volume = (Width(2) × Length)/2), mice were treated daily, for a total of either 1, 3 or 7 days, by oral gavage with AZD2014 (20 or 25 mg/kg/day) or vehicle control. Mice were monitored daily for body weight or other adverse effects.

### Biodistribution and imaging

When the tumours reached approximately 100–200 mm^3^, mice were anaesthetised with an isoflurane/O_2_ mixture (1.5–2.0% v/v) and intravenously injected with either ^18^F-mFBG (~ 7.5 MBq) or ^123^I-mIBG (~ 20 MBq). Mice were then imaged using an Albira PET/SPECT/CT scanner (Bruker, Coventry, UK). Whole body PET static images were acquired after 1 and 4 h post-injection (p.i.) for 10 min with an energy window of 358 to 664 keV followed by CT acquisition. Acquisition, reconstruction and image analysis were performed as described previously^66^. Following the final scan, the mice were sacrificed and the major organs were excised, weighed and their associated radioactivity was measured using a γ-counter. Biodistribution and image quantification were expressed as a percentage of injected dose per gram of tissue (%ID/g), whereby 1 cm^3^ on PET acquisitions were assumed to equal 1 g (n ≥ 3 mice ± SD).

### Autoradiography

Dissected tumours were set in Tissue-Tek optimal-cutting-temperature compound (Sakura, Torrance, California, USA) and snap-frozen in liquid nitrogen. The tumours were then sectioned to a thickness of 10 µm using a cytomicrotome (Thermo Fisher Scientific, Loughborough, UK) and mounted on slides, that were exposed to X-ray film for 24 h and scanned using a Typhoon FLA 7000 phosphorimager (GE Healthcare Life Sciences, Amersham, UK).

### Immunohistochemistry

Formalin-fixed (10% v/v) paraffin-embedded tumours were sectioned to a thickness of 5 µm. Tumour sections underwent heat-induced epitope retrieval with a citric acid based antigen unmasking solution (H3300, Vector Laboratories, Peterborough, UK). The primary NET-1 antibody (AMT-002, Alomone Labs, Jerusalem, Israel), p-S6^S240/244^ and p-4EBP1^T37/46^ were added and incubated overnight at 4 °C. Endogenous peroxidases were blocked (2% H_2_O_2_ in methanol, 10 min, RT) before incubation with a secondary HRP-conjugated antibody. For chromogen development, slides were processed using a diaminobenzidine (DAB)-peroxidase substrate kit (4 min, Immpact DAB, SK-4105, Vector Laboratories, Peterborough, UK). Samples were counterstained with Gills III haematoxylin and mounted with a coverslip. Digital images were captured on a Nanozoomer XR (Hamamatsu Photonics, Hamamatsu, Japan).

### Statistics

Data were presented as mean ± SD, unless otherwise stated. Statistical analyses were performed using two-way ANOVA with multiple comparisons using Tukey’s post-hoc test: *p < 0.05, **p < 0.01, ***p < 0.001, ****p < 0.0001, using GraphPad Prism software (v.8, La Jolla, CA, USA)**.**

## Supplementary information

Supplementary information.

## References

[1] Berthold F, Spix C, Kaatsch P, Lampert F (2017). Incidence, survival, and treatment of localized and metastatic neuroblastoma in Germany 1979–2015. Paediatr. Drugs.

[2] Childhood Cancer Statistics, England Annual report 2018. *National Cancer Registration and Analysis Service* (2018). Accessed http://ncin.org/home (2019).

[3] Matthay KK, George RE, Yu AL (2012). Promising therapeutic targets in neuroblastoma. Clin. Cancer Res..

[4] Maris JM, Hogarty MD, Bagatell R, Cohn SL (2007). Neuroblastoma. Lancet.

[5] Smith V, Foster J (2018). High-risk neuroblastoma treatment review. Children (Basel, Switzerland).

[6] Pinto NR (2015). Advances in risk classification and treatment strategies for neuroblastoma. J. Clin. Oncol..

[7] Brodeur GM, Bagatell R (2014). Mechanisms of neuroblastoma regression. Nat. Rev. Clin. Oncol..

[8] Barone G, Anderson J, Pearson AD, Petrie K, Chesler L (2013). New strategies in neuroblastoma: Therapeutic targeting of MYCN and ALK. Clin. Cancer Res..

[9] Vaughan L (2016). Inhibition of mTOR-kinase destabilizes MYCN and is a potential therapy for MYCN-dependent tumors. Oncotarget.

[10] Valentijn LJ (2012). Functional MYCN signature predicts outcome of neuroblastoma irrespective of MYCN amplification. Proc. Natl. Acad. Sci. USA.

[11] Mei H, Wang Y, Lin Z, Tong Q (2013). The mTOR signaling pathway in pediatric neuroblastoma. Pediatr. Hematol. Oncol..

[12] Opel D, Poremba C, Simon T, Debatin K-M, Fulda S (2007). Activation of Akt predicts poor outcome in neuroblastoma. Can. Res..

[13] Kogner JIJ (2007). Inhibitors of mammalian target of rapamycin downregulate MYCN protein expression and inhibit neuroblastoma growth. Oncogene.

[14] Zhang H (2015). mTOR ATP-competitive inhibitor INK128 inhibits neuroblastoma growth via blocking mTORC signaling. Apoptosis.

[15] Xu D-Q (2018). Anti-tumor effect of AZD8055 against neuroblastoma cells in vitro and in vivo. Exp. Cell Res..

[16] Naing A (2012). Safety, tolerability, pharmacokinetics and pharmacodynamics of AZD8055 in advanced solid tumours and lymphoma. Br. J. Cancer.

[17] Ohtsu A (2013). Everolimus for previously treated advanced gastric cancer: Results of the randomized, double-blind, phase III GRANITE-1 study. J. Clin. Oncol..

[18] Magaway C, Kim E, Jacinto E (2019). Targeting mTOR and metabolism in cancer: Lessons and innovations. Cells.

[19] Hu YF, Caron MG, Sieber-Blum M (2009). Norepinephrine transport-mediated gene expression in noradrenergic neurogenesis. BMC Genomics.

[20] DuBois SG, Matthay KK (2008). Radiolabeled metaiodobenzylguanidine for the treatment of neuroblastoma. Nucl. Med. Biol..

[21] Sharp SE, Trout AT, Weiss BD, Gelfand MJ (2016). MIBG in neuroblastoma diagnostic imaging and therapy. Radiographics.

[22] Howard JP (2005). Tumor response and toxicity with multiple infusions of high dose 131I-MIBG for refractory neuroblastoma. Pediatr. Blood Cancer.

[23] Sisson JC (1988). Toxicity from treatment of neuroblastoma with 131I-meta-iodobenzylguanidine. Eur. J. Nucl. Med..

[24] Matthay KK (2012). Dose escalation study of no-carrier-added 131I-metaiodobenzylguanidine for relapsed or refractory neuroblastoma: New approaches to neuroblastoma therapy consortium trial. J. Nucl. Med..

[25] Mairs, R. J. *et al.* No-carrier-added iodine-131-MIBG: Evaluation of a therapeutic preparation. *J. Nucl. Med.* **36**, 1088–1095 (1995). Retrieved from http://jnm.snmjournals.org/ (2020). 7769433

[26] DuBois SG (2015). Phase I/II study of (131)I-MIBG with vincristine and 5 days of irinotecan for advanced neuroblastoma. Br. J. Cancer.

[27] 27Houghton, P. J. & Santana, V. M. Clinical trials using irinotecan. *J. Pediatr. Hematol./Oncol.* **24**, 84–85 (2002). Retrieved from https://journals.lww.com/jpho-online/pages/default.aspx (2020). 10.1097/00043426-200202000-00002 11990709

[28] Chen C-S, Weng S-C, Tseng P-H, Lin H-P, Chen C-S (2005). Histone acetylation-independent effect of histone deacetylase inhibitors on Akt through the reshuffling of protein phosphatase 1 complexes. J. Biol. Chem..

[29] Robertson SD (2010). Insulin reveals Akt signaling as a novel regulator of norepinephrine transporter trafficking and norepinephrine homeostasis. J. Neurosci..

[30] Siuta MA (2010). Dysregulation of the norepinephrine transporter sustains cortical hypodopaminergia and schizophrenia-like behaviors in neuronal rictor null mice. PLoS Biol..

[31] Pandit-Taskar N (2018). Biodistribution and dosimetry of (18)F-meta-fluorobenzylguanidine: A first-in-human PET/CT imaging study of patients with neuroendocrine malignancies. J. Nucl..

[32] Cistaro A (2015). 124I-MIBG: A new promising positron-emitting radiopharmaceutical for the evaluation of neuroblastoma. Nucl. Med. Rev. Cent. East. Eur..

[33] Aboian M (2020). 124I-MIBG PET-CT to monitor metastatic disease in children with relapsed neuroblastoma. J. Nucl. Med..

[34] Huang S-Y (2015). Patient-specific dosimetry using pretherapy [^124^I]m-iodobenzylguanidine ([^124^I]mIBG) dynamic PET/CT imaging before [^131^I]mIBG targeted radionuclide therapy for neuroblastoma. Mol. Imaging Biol..

[35] Pentlow KS, Graham MC, Lambrecht RM, Cheung NK, Larson SM (1991). Quantitative imaging of I-124 using positron emission tomography with applications to radioimmunodiagnosis and radioimmunotherapy. Med. Phys..

[36] Kuker R, Sztejnberg M, Gulec S (2017). I-124 imaging and dosimetry. I-124 Görüntüleme ve Dozimetri. Mol. Imaging Radionucl. Ther..

[37] Garg PK, Garg S, Zalutsky MR (1994). Synthesis and preliminary evaluation of para- and meta-[18F]fluorobenzylguanidine. Nucl. Med. Biol..

[38] Zhang H (2014). Imaging the norepinephrine transporter in neuroblastoma: A comparison of [18F]-MFBG and 123I-MIBG. Clin. Cancer Res..

[39] Rotstein BH (2016). Mechanistic studies and radiofluorination of structurally diverse pharmaceuticals with spirocyclic iodonium(iii) ylides. Chem. Sci..

[40] Hu B (2015). A practical, automated synthesis of meta-[(18)F]fluorobenzylguanidine for clinical use. ACS Chem. Neurosci..

[41] Makaravage KJ, Brooks AF, Mossine AV, Sanford MS, Scott PJH (2016). Copper-mediated radiofluorination of arylstannanes with [18F]KF. Org. Lett..

[42] Preshlock S (2016). Enhanced copper-mediated 18F-fluorination of aryl boronic esters provides eight radiotracers for PET applications. Chem. Commun..

[43] More SS (2011). Vorinostat increases expression of functional norepinephrine transporter in neuroblastoma in vitro and in vivo model systems. Clin. Cancer Res..

[44] Li H, Ma SK, Hu XP, Zhang GY, Fei J (2001). Norepinephrine transporter (NET) is expressed in cardiac sympathetic ganglia of adult rat. Cell Res..

[45] Beltran H (2014). The N-myc oncogene: Maximizing its targets, regulation, and therapeutic potential. Mol. Cancer Res..

[46] Huang M, Weiss WA (2013). Neuroblastoma and MYCN. Cold Spring Harb. Perspect. Med..

[47] Smith JR (2016). Novel pharmacodynamic biomarkers for MYCN protein and PI3K/AKT/mTOR pathway signaling in children with neuroblastoma. Mol. Oncol..

[48] Becher OJ (2017). A phase I study of perifosine with temsirolimus for recurrent pediatric solid tumors. Pediatr. Blood Cancer.

[49] Pearson ADJ (2016). A phase 1 study of oral ridaforolimus in pediatric patients with advanced solid tumors. Oncotarget.

[50] Dubois SG (2012). Evaluation of norepinephrine transporter expression and metaiodobenzylguanidine avidity in neuroblastoma: A report from the Children's Oncology Group. Int. J. Mol. Imaging.

[51] DuBois SG (2017). MIBG avidity correlates with clinical features, tumor biology, and outcomes in neuroblastoma: A report from the Children's Oncology Group. Pediatr. Blood Cancer..

[52] Schmidt M, Simon T, Hero B, Schicha H, Berthold F (2008). The prognostic impact of functional imaging with (123)I-mIBG in patients with stage 4 neuroblastoma >1 year of age on a high-risk treatment protocol: Results of the German Neuroblastoma Trial NB97. Eur. J. Cancer.

[53] Matthay KK (2003). Correlation of early metastatic response by 123I-metaiodobenzylguanidine scintigraphy with overall response and event-free survival in stage IV neuroblastoma. J. Clin. Oncol..

[54] Ady N (1995). A new 123I-MIBG whole body scan scoring method—Application to the prediction of the response of metastases to induction chemotherapy in stage IV neuroblastoma. Eur. J. Cancer.

[55] Messina JA (2006). Evaluation of semi-quantitative scoring system for metaiodobenzylguanidine (mIBG) scans in patients with relapsed neuroblastoma. Pediatr. Blood Cancer.

[56] Matthay KK (2010). Criteria for evaluation of disease extent by 123I-metaiodobenzylguanidine scans in neuroblastoma: A report for the International Neuroblastoma Risk Group (INRG) Task Force. Br. J. Cancer.

[57] Preshlock S, Tredwell M, Gouverneur V (2016). (18)F-labeling of arenes and heteroarenes for applications in positron emission tomography. Chem. Rev..

[58] Preshlock S (2016). Enhanced copper-mediated (18)F-fluorination of aryl boronic esters provides eight radiotracers for PET applications. Chem. Commun. (Camb.).

[59] Pike (2015). AZD2014, an inhibitor of mTORC1 and mTORC2, is highly effective in ER+ breast cancer when administered using intermittent or continuous schedules. Mol. Cancer Ther..

[60] Chresta CM (2010). AZD8055 is a potent, selective, and orally bioavailable ATP-competitive mammalian target of rapamycin kinase inhibitor with in vitro and in vivo antitumor activity. Can. Res..

[61] Zormpas-Petridis K (2020). Noninvasive MRI native T1 mapping detects response to MYCN-targeted therapies in the Th-MYCN model of neuroblastoma. Cancer Res..

[62] Wong Te Fong AC (2017). Evaluation of the combination of the dual m-TORC1/2 inhibitor vistusertib (AZD2014) and paclitaxel in ovarian cancer models. Oncotarget.

[63] Faes S, Santoro T, Demartines N, Dormond O (2017). Evolving significance and future relevance of anti-angiogenic activity of mTOR inhibitors in cancer therapy. Cancers (Basel).

[64] Kramer-Marek G, Kiesewetter DO, Capala J (2009). Changes in HER2 expression in breast cancer xenografts after therapy can be quantified using PET and (18)F-labeled affibody molecules. J. Nucl. Med..

[65] Robinson (2010). Guidelines for the welfare and use of animals in cancer research. Br. J. Cancer.

[66] Kramer-Marek CDM (2018). HER3-mediated resistance to Hsp90 inhibition detected in breast cancer xenografts by affibody-based PET imaging. Clin. Cancer Res..

