# Supplementary material for: 18F-meta-fluorobenzylguanidine (18F-mFBG) to monitor changes in norepinephrine transporter expression in response to therapeutic intervention in neuroblastoma models
Source: Sci Rep. 2020 Dec 1;10:20918. doi: 10.1038/s41598-020-77788-3 (PMC7708446; doi:10.1038/s41598-020-77788-3)
Supplement: Supplementary file 1 — Supplementary information. [file 41598_2020_77788_MOESM1_ESM.docx]

**Supplementary Material**

**Title:** ^18^F-meta-fluorobenzylguanidine (^18^F-mFBG) to monitor changes in norepinephrine transporter expression in response to therapeutic intervention in neuroblastoma models.

**Authorship:** Stephen Turnock^1^, David R Turton^2^, Carlos Daniel Martins^1^, Louis Chesler^3^, Thomas C. Wilson^4^, Véronique Gouverneur^4^, Graham Smith^2^, Gabriela Kramer-Marek*^1^.

^1^ Preclinical Molecular Imaging, Division of Radiotherapy and Imaging, The Institute of Cancer Research, 123 Old Brompton Road, London SW7 3RP, UK.

^2^ PET Radiochemistry, Division of Radiotherapy and Imaging, The Institute of Cancer Research, 123 Old Brompton Road, London SW7 3RP, UK.

^3^ Division of Clinical Studies, The Institute of Cancer Research, 123 Old Brompton Road, London SW7 3RP, UK.

^4^ Department of Chemistry, University of Oxford, 12 Mansfield Road, Oxford, OX1 3TA, UK.

***Corresponding author**: Gabriela Kramer-Marek; gabriela.kramer-marek@icr.ac.uk; The Institute of Cancer Research, 123 Old Brompton Road, London SW7 3RP, UK; +44(0)20-8722-4124. https://orcid.org/0000-0002-8053-3880

**First author:** Stephen Turnock; stephen.turnock@icr.ac.uk; The Institute of Cancer Research, 123 Old Brompton Road, London SW7 3RP, UK; +44(0)20-8722-4611. https://orcid.org/0000-0002-1959-3569

**^18^F-mFBG production:**

^18^F fluoride was produced on a GE PETrace cyclotron by 16 MeV irradiation of an enriched [^18^O]H_2_O target, supplied by Alliance Medical UK (Warwick, UK). ^18^F-fluoride (~16 GBq) in water was delivered to the Trasis module and loaded on a Sep-Pak Accell Plus QMA Plus Light Cartridge (46 mg sorbent, Waters, Elstree, UK). The ^18^F fluoride was eluted using a 1 ml solution of Krypofix 222 (2.5 mg, Sigma-Aldrich, Gillingham, UK), an aqueous solution of potassium carbonate (0.1 mL, 5 mg/mL, Sigma-Aldrich, Gillingham, UK) and acetonitrile (0.9 ml, Thermo Fisher Scientific, Loughborough, UK). The eluted solution was dried at 125 °C under a stream of nitrogen with gradually increasing applied vacuum. A reaction mixture consisting of tetrakis pyridine copper triflate (5 mg (7.4 µMol), Sigma-Aldrich, Gillingham, UK) and (tert-butyl-N-[(1Z)-{bis[(tertbutoxy)carbonyl]amino}({[(tert-butoxy)carbonyl]({[3-(tetramethyl-1,3,2-dioxaborolan-2-yl)phenyl]methyl})amino})methylidene] carbamate) (10 mg (14.8 µMol); provided by Professor V. Gouverneur, Oxford University, Oxford, UK)^35^ in anhydrous DMF (0.5 ml, Sigma-Aldrich, Gillingham, UK) was added to the dry ^18^F-fluoride and heated for 20 min at 120 °C. A water flush was used to remove unreacted ^18^F from the reaction mixture by solid phase extraction (SPe) using an Oasis Plus short HLB cartridge (225 mg sorbent; Waters, Elstree, UK). The crude product was eluted with acetone (2 ml, Sigma-Aldrich, Gillingham, UK), and subsequently dried under a nitrogen flow. The BOC protecting groups were then removed by incubating the solution in hydriodic acid (57%, 400 µl; Sigma-Aldrich, Gillingham, UK) at 125 °C for 10 min. The crude product was dissolved in water (9 ml) and purified by semi-preparative HPLC on a Luna C18 column (7.8 × 250 mm, 10 µm, Phenomenex, Macclesfield, UK), with isocratic elution of 10% ethanol / 90% of 0.1% aqueous phosphoric acid (H_3_PO_4_, Sigma-Aldrich, Gillingham, UK) at a flow rate of 3 mL/min. The column output was monitored for radioactivity and UV absorption at 254 nm. The purified product was collected into a vial containing water (20 ml) and then concentrated by weak cation exchange SPE (Strata X CW resin, 30 mg sorbent; Phenomenex, Macclesfield, UK). The product was eluted using 0.1% aqueous H_3_PO_4_ in ethanol (1:4 v/v), dried under a nitrogen flow, and then dissolved in the desired volume of water. The chemical and radiochemical purity, and the molar activity were determined by RP-HPLC analysis using an Agilent Infinity 1260 quaternary pump system equipped with a 1260 variable wavelength UV detector (Agilent Technologies, Didcot, UK) fitted with a Luna 5 µm C18 column (150 × 4.6 mm; Phenomenex, Macclesfield, UK). An isocratic elution with 95% of 0.1% aqueous H_3_PO_4_ / 5% acetonitrile as mobile phase was used at a flow rate of 1 mL/min. Elution profiles were recorded using Laura software (Lablogic, Sheffield, UK). The UV absorbance was recorded at 254 nm. The radioactivity of the eluate was monitored using a NaI radiodetector (Lablogic, Sheffield, UK). Retention times (Rt) are expressed as minutes:seconds (min:sec). An injection of a reference solution of mFBG was performed for the determination of the product molar activity. The purified product was achieved with a radio-chemical purity of >98% verified by RP-HPLC: Average retention time: 06:51 min:sec; RCY (decay corrected): 6.0 ± 1.4%; molar activity: 59.0 ± 35.9 GBq/µmol (n = 10).


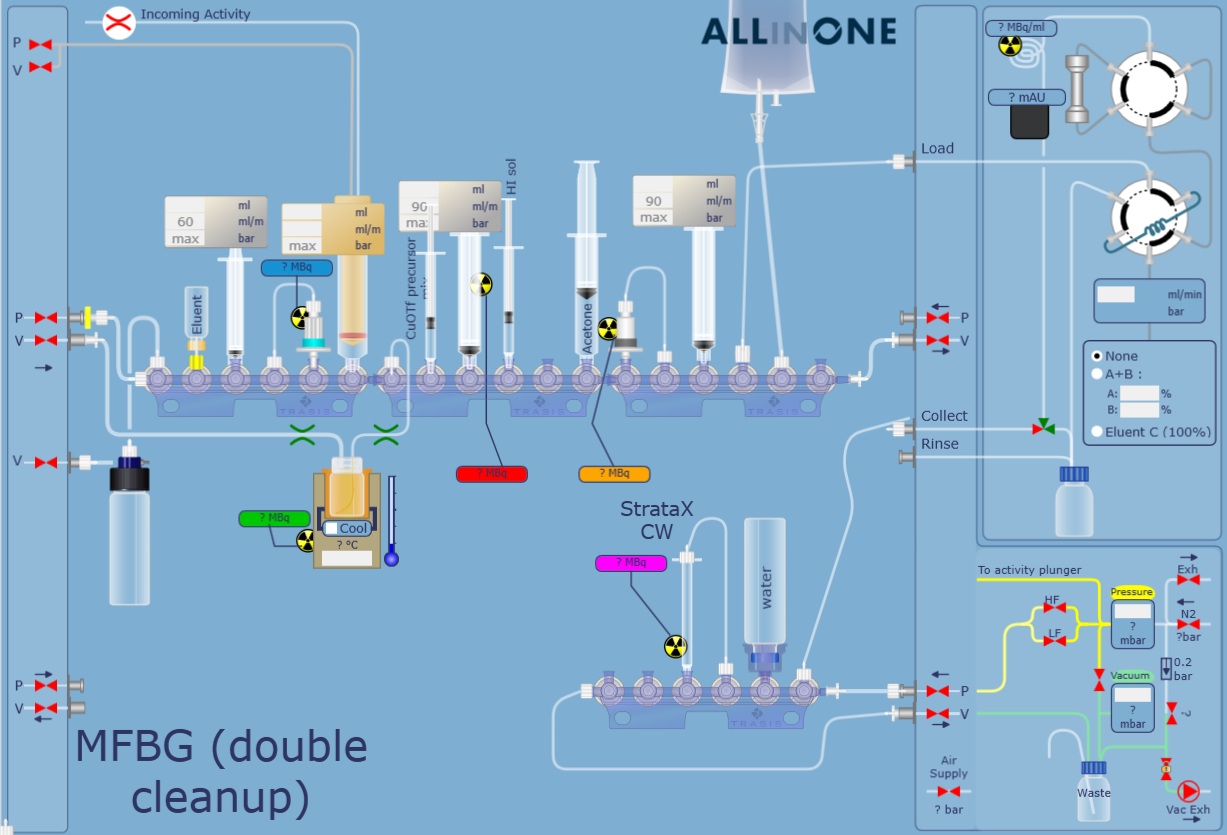


**Supplementary Figure 1:** Schematic of Trasis AiO module setup for ^18^F-mFBG production. Printed with permission of Trasis Pharmacy Instruments, Belgium.


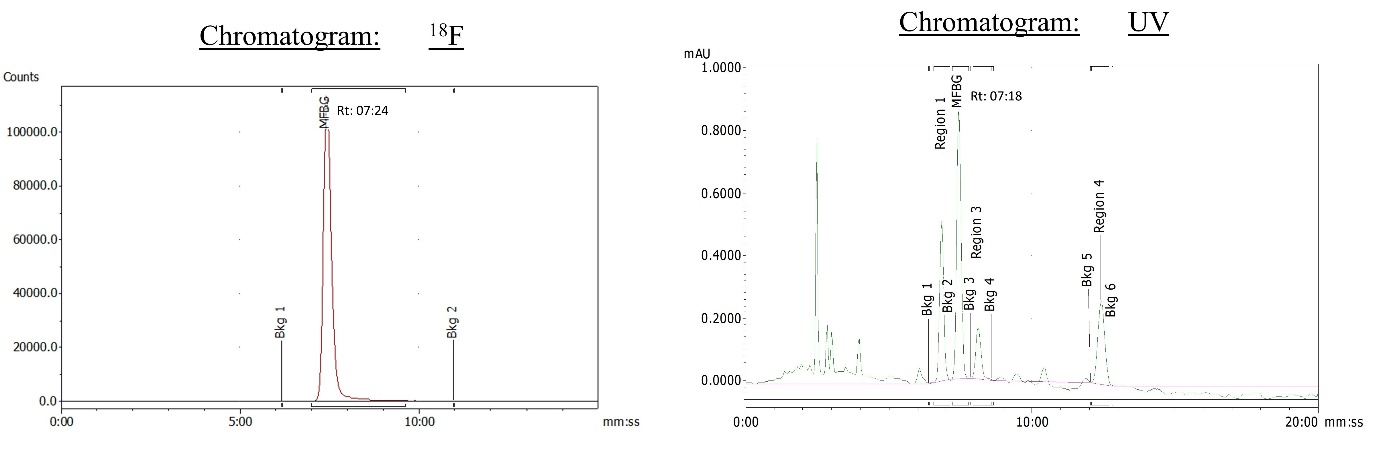


**Supplementary Figure 2:** Example HPLC trace of ^18^F-mFBG synthesis validation. Average retention times (Rt; mm:ss) were 06:51 in the UV channel (range: 06:19–07:23) and 06:57 in the ^18^F channel (range: 06:23–07:29).


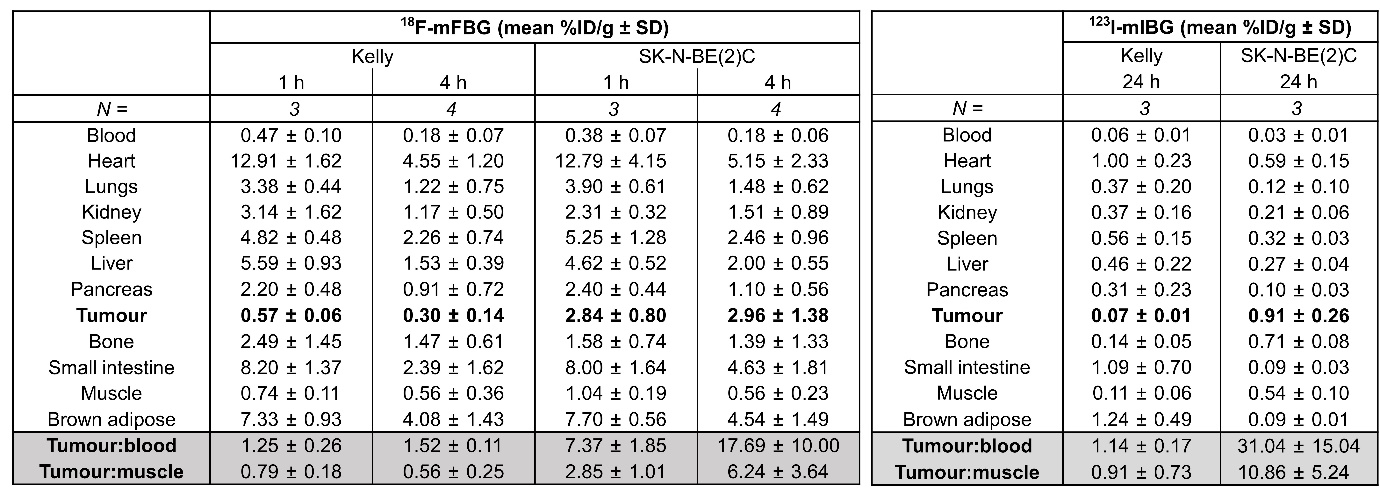


**Supplementary Table 1:** Biodistribution of ^18^F-mFBG and ^123^I-mIBG 1, 4 and 24 h after injection of mice bearing SK-N-BE(2)C or Kelly xenografts. Data presented as mean ± SD, n ≥ 3 per group.


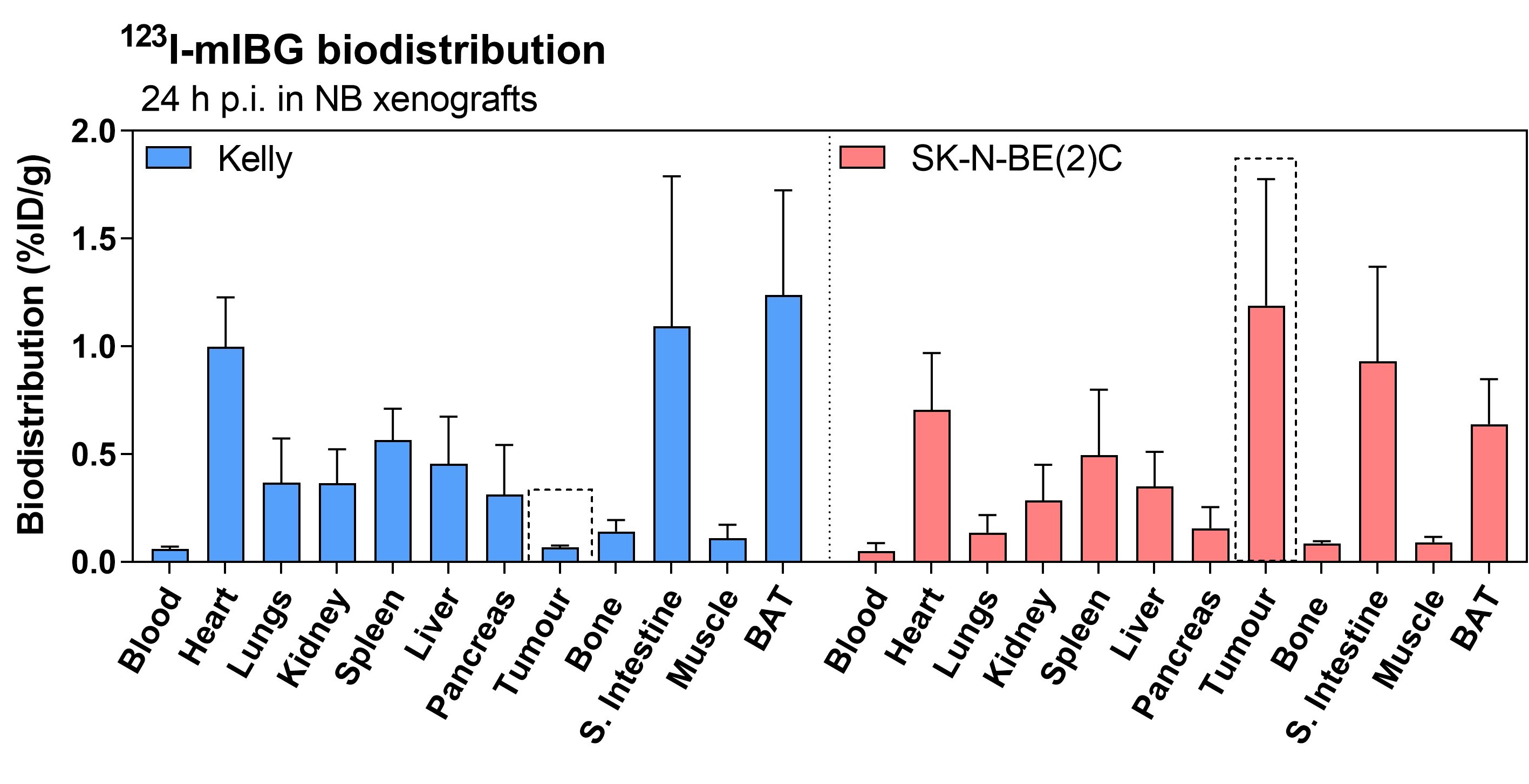


**Supplementary Figure 3**: ^123^I-mIBG biodistribution in Kelly and SK-N-BE(2)C tumour bearing mice, 24 h p.i. Data presented as mean ± SD, n = 3 per group. Graph is generated using GraphPad Prism (v 8.4.1), https://www.graphpad.com.


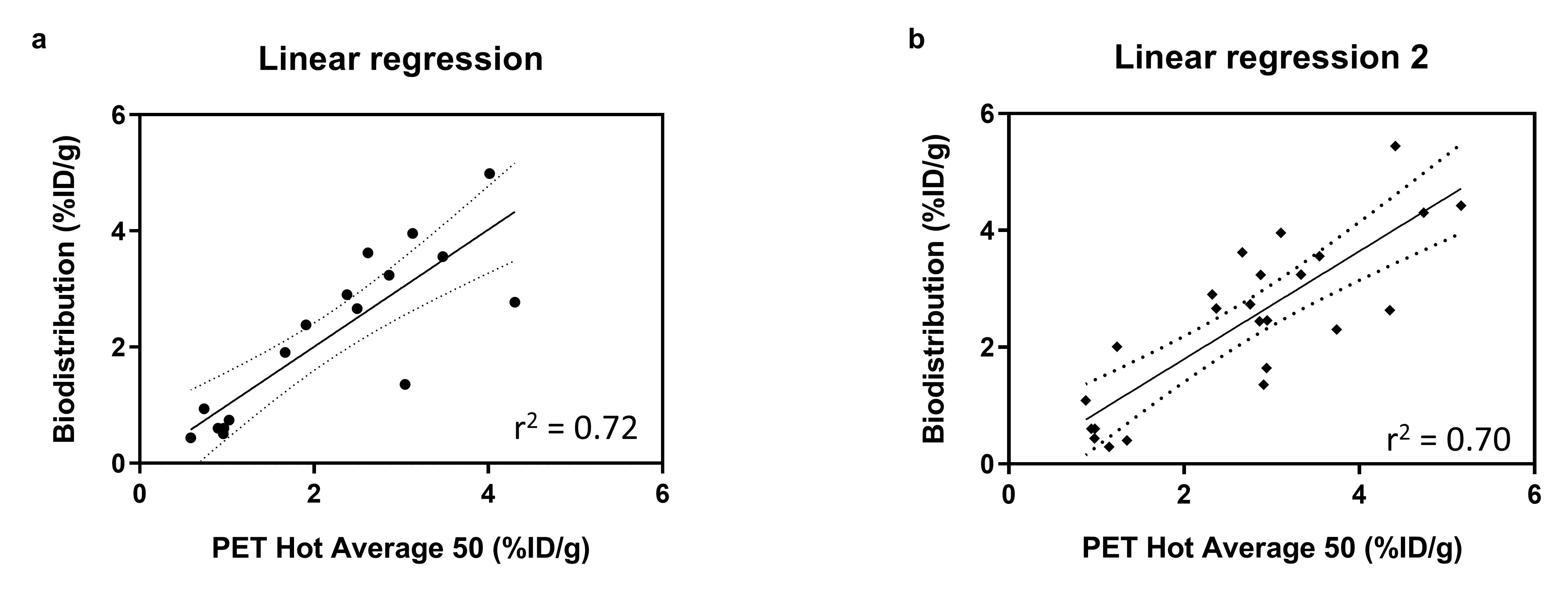


**Supplementary Figure 4: a)** Linear regression of PET quantification (average of hottest 50 voxels in VOI) against biodistribution of mice bearing SK-N-BE(2)C or Kelly tumour xenografts. **b)** Linear regression fit of PET quantification (average of hottest 50 voxels in VOI) against biodistribution of SK-N-BE(2)C or Kelly tumour bearing mice treated with AZD2014. Graphs are generated using GraphPad Prism (v 8.4.1), https://www.graphpad.com.


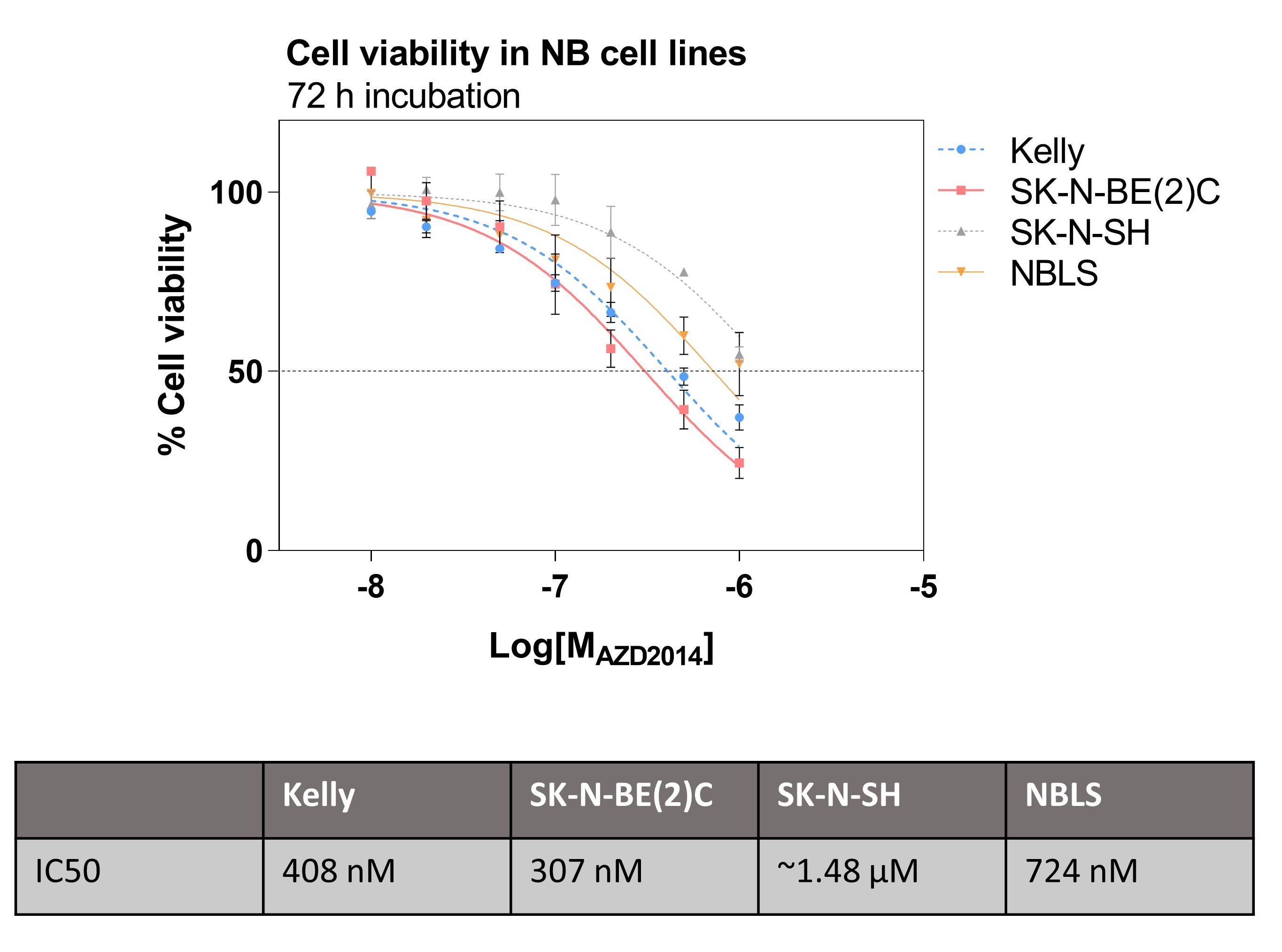


**Supplementary Figure 5:** NB cell viability after 72 h treatment with AZD2014 (0–1 µM) analysed by Celltiter Glo assay. Data presented as mean ± SEM, n ≥ 3 per group, performed in triplicate. Graph is generated using GraphPad Prism (v 8.4.1), https://www.graphpad.com.

**
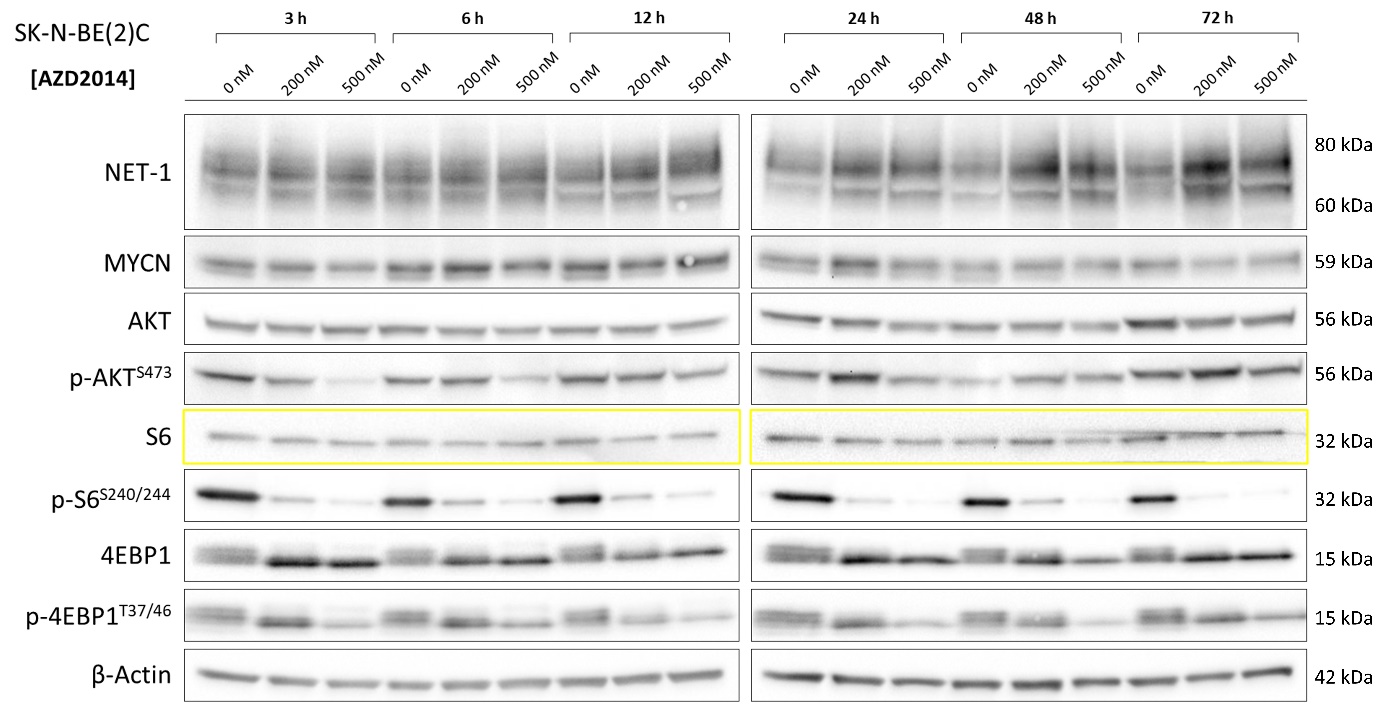
**

**Supplementary Figure 6:** Western blot of SK-N-BE(2)C cells treated with AZD2014 (0–500 nM) for 3–72 h. Yellow outline indicates a second membrane of the same lysates for better protein signal acquisitions.


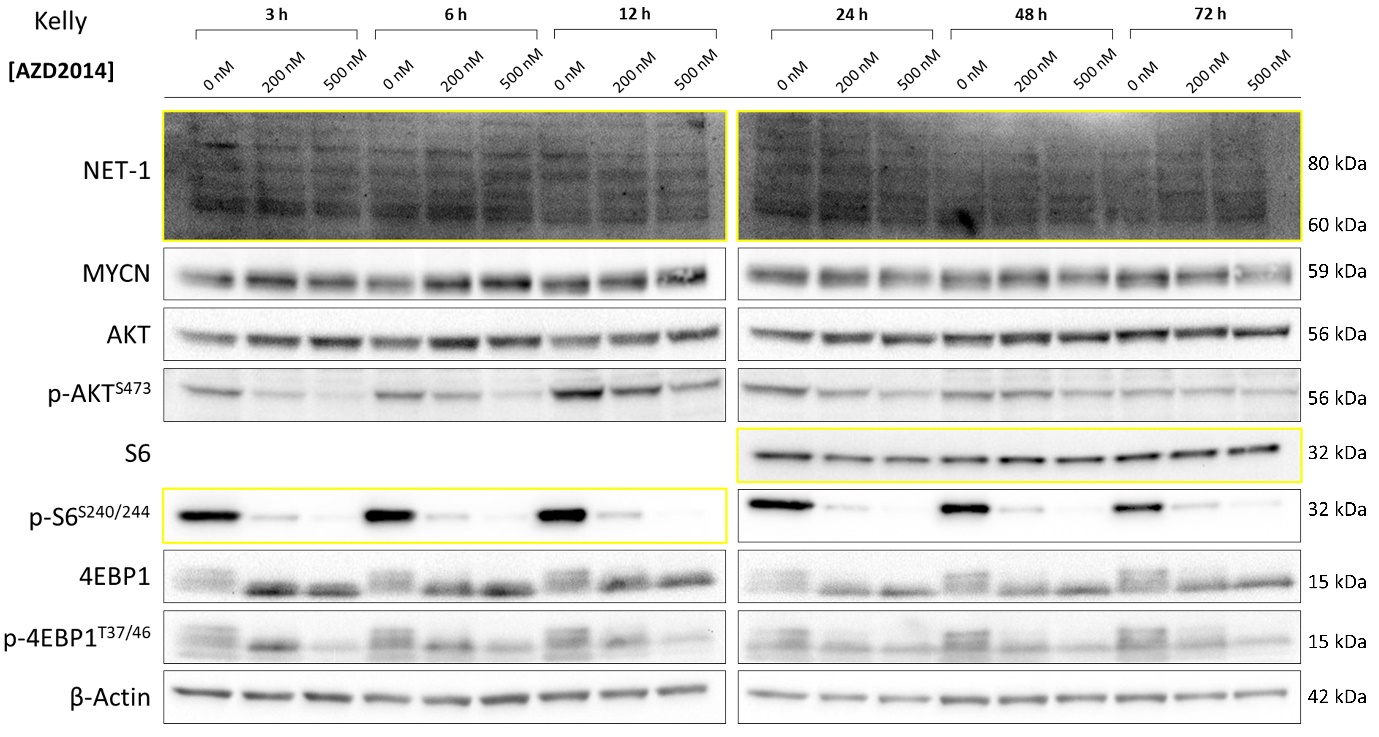


**Supplementary Figure 7:** Western blot of Kelly cells treated with AZD2014 (0–500 nM) for 3–72 h. Yellow outline indicates a second membrane of the same lysates for better protein signal acquisitions.


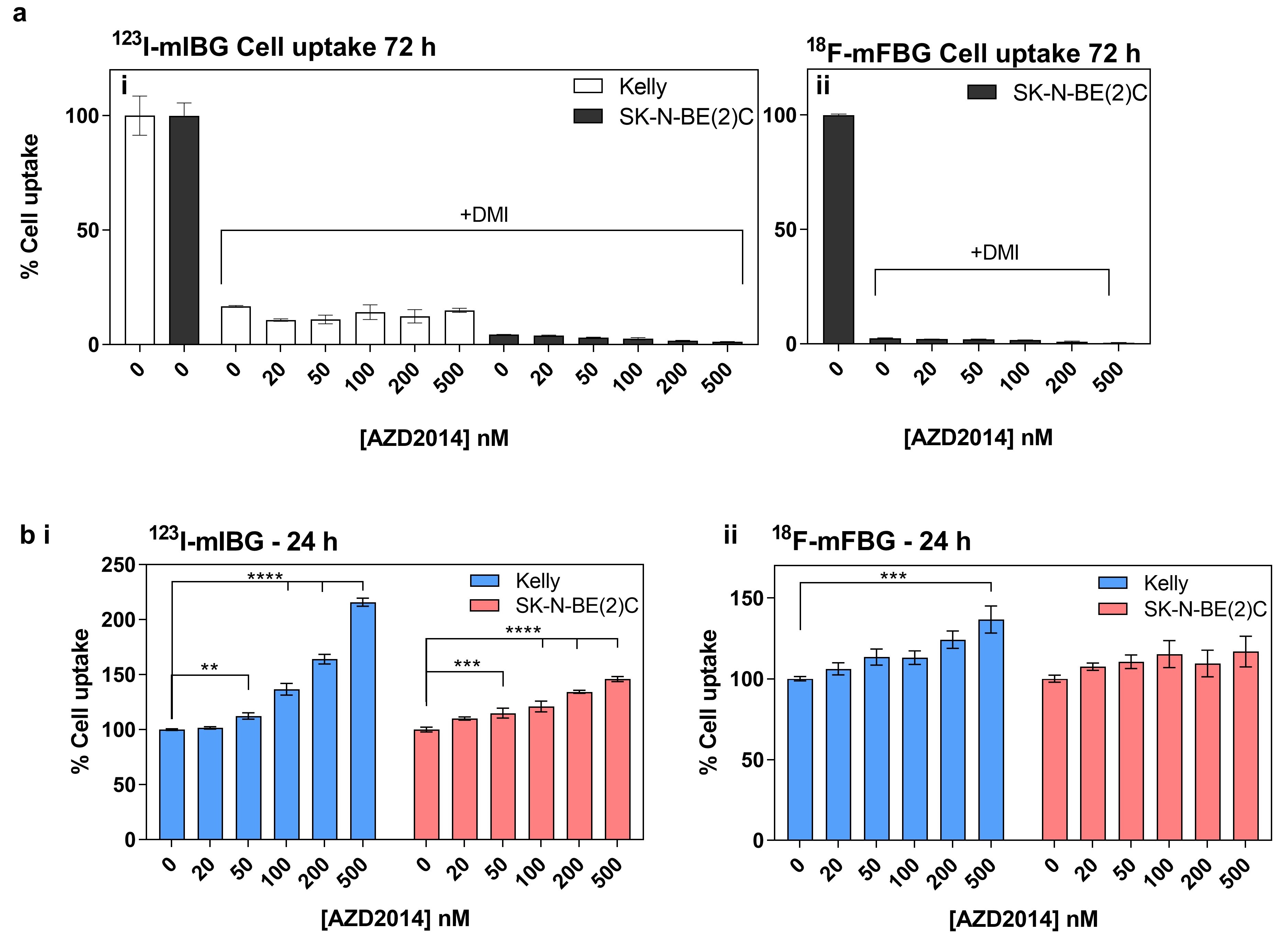


**Supplementary Figure 8: a)** Cell associate tracer uptake in Kelly and SK-N-BE(2)C cells treated with AZD2014 (72 h; 0–500 nM) and blocked with 50 µM desipramine (DMI) with i) ^123^I-mIBG and ii) ^18^F-mFBG. **b)** Cell associated ^123^I-mIBG and ^18^F-mFBG tracer uptake in cells treated with AZD2014 (0–500 nM) for 24 h. Data presented as mean ± SD, n ≥ 1 per group, performed in triplicate. Graphs are generated using GraphPad Prism (v 8.4.1), https://www.graphpad.com.

**Supplementary Figure 9: a)** Change in body weight in animals treated with AZD2014 20mg/kg/day (n ≥ 6) or 25mg/kg/day (n = 6) or vehicle control (n ≥ 6) **b)** Relative tumour volume of SK-N-BE(2)C xenografts during treatment with AZD2014 20mg/kg/day (n ≥ 6) or vehicle (n ≥ 6). **c)** Relative tumour volume of Kelly xenografts during treatment with AZD2014 25mg/kg/day (n =3) or vehicle (n = 3). Graphs are generated using GraphPad Prism (v 8.4.1), https://www.graphpad.com.


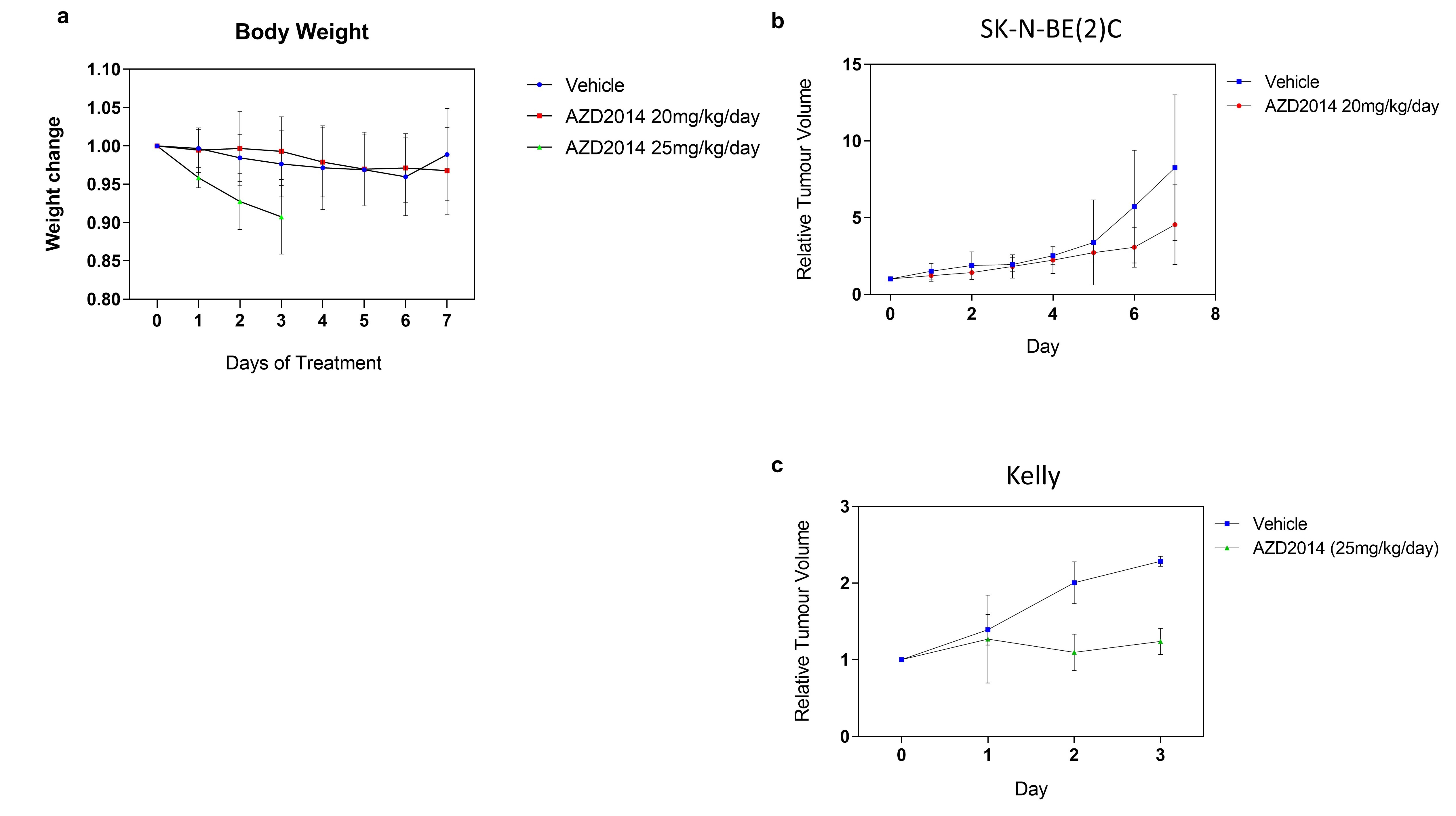

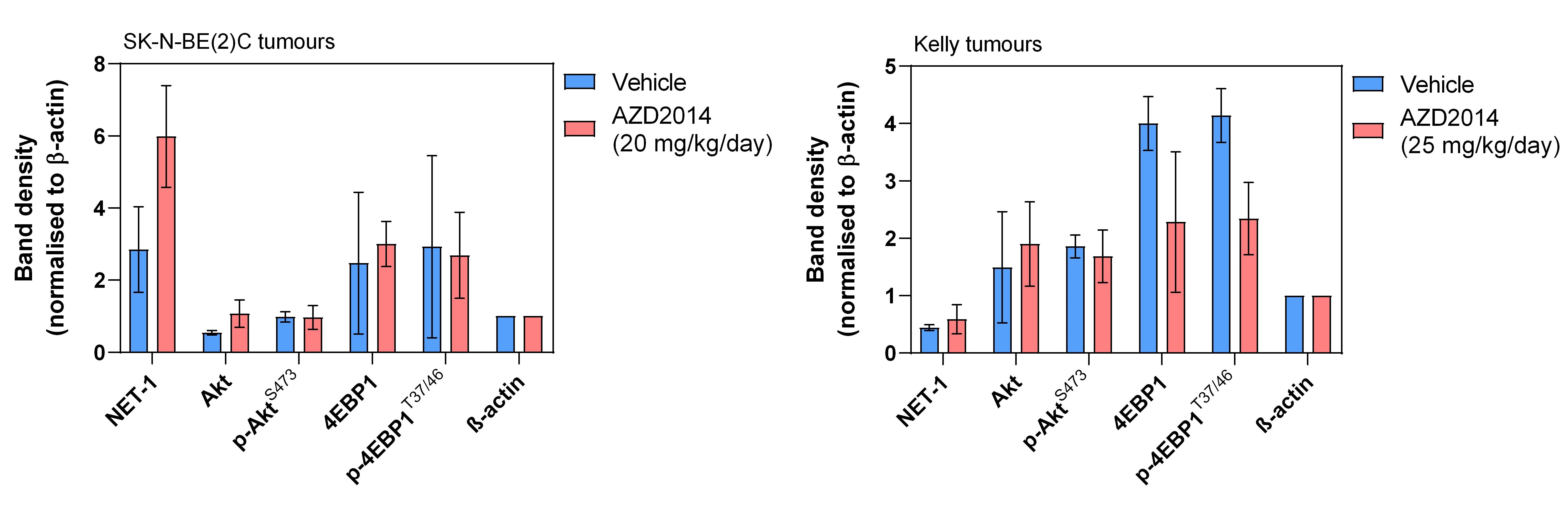


**Supplementary Figure 10:** Normalised band density of tumour protein lysate from Western blot analysis (seen in Fig 5) for SK-N-BE(2)C (left) and Kelly (right) of animals treated with AZD2014 (20 or 25 mg/kg/day) or vehicle. Differences in band density were compared using a Student’s T-test with Holm-Sidak test for significance. Graphs are generated using GraphPad Prism (v 8.4.1), https://www.graphpad.com.

**Supplementary Western blots:** Immunoblots with membrane photo overlay and band density. Membranes were cut with a scalpel before probing with the detection antibody according to the protein markers to encapsulate all regions of interest as determined by the antibody reference sheet provided by the supplier. Red dashed boxes indicate the area used for the WB presented. Yellow boxes indicate a second membrane of the same lysates. A control band was included for all membranes.

**For Figure 1b**


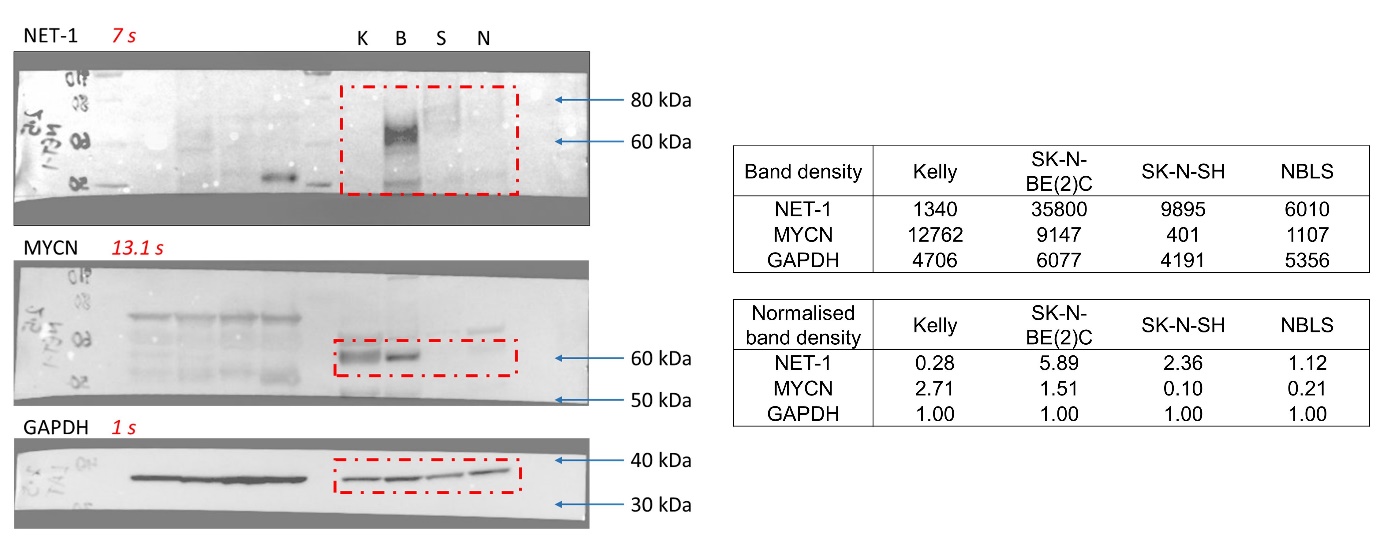


**WB1:** Immunoblots of neuroblastoma cell lysates with membrane photo overlay and band density for Figure 1b. Red dashed boxes indicate the area used for the WB presented. Band density and intensity ratio (normalised to GAPDH) are included in the tables to the right.

**For Figure 3c/3d**


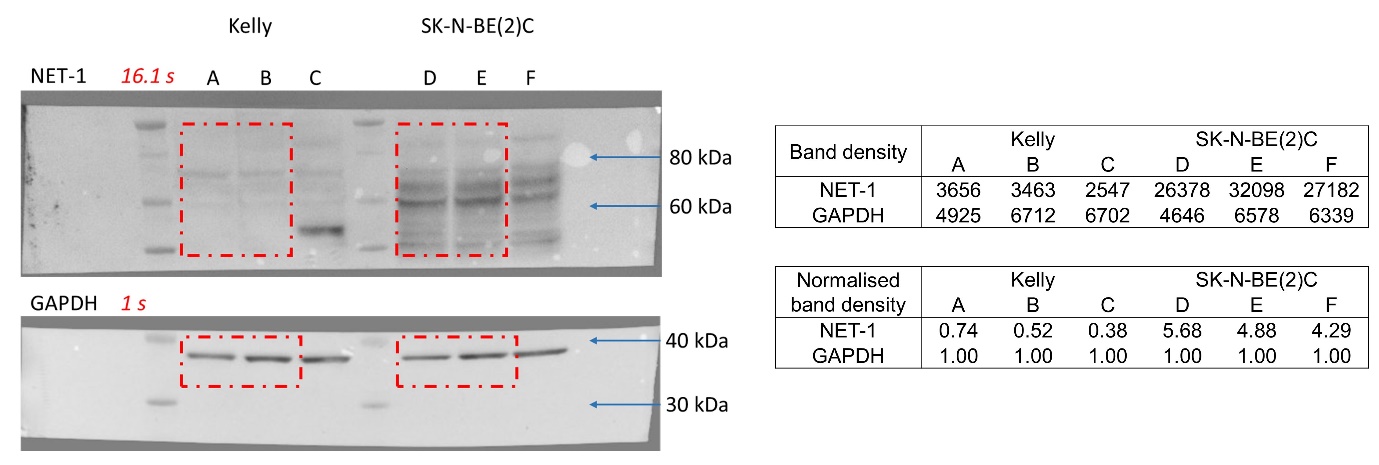


**WB2:** Immunoblots of Kelly and SK-N-BE(2)C *ex vivo* tumour lysates with membrane photo overlay and band density for Figure 3c (lanes A and B) and Figure 3d (lanes D and E). Red dashed boxes indicate the area used for the WB presented. Exposure time is indicated in red italics. Band density and intensity ratio (normalised to GAPDH) are included in the tables to the right.

**For Figure 5b**


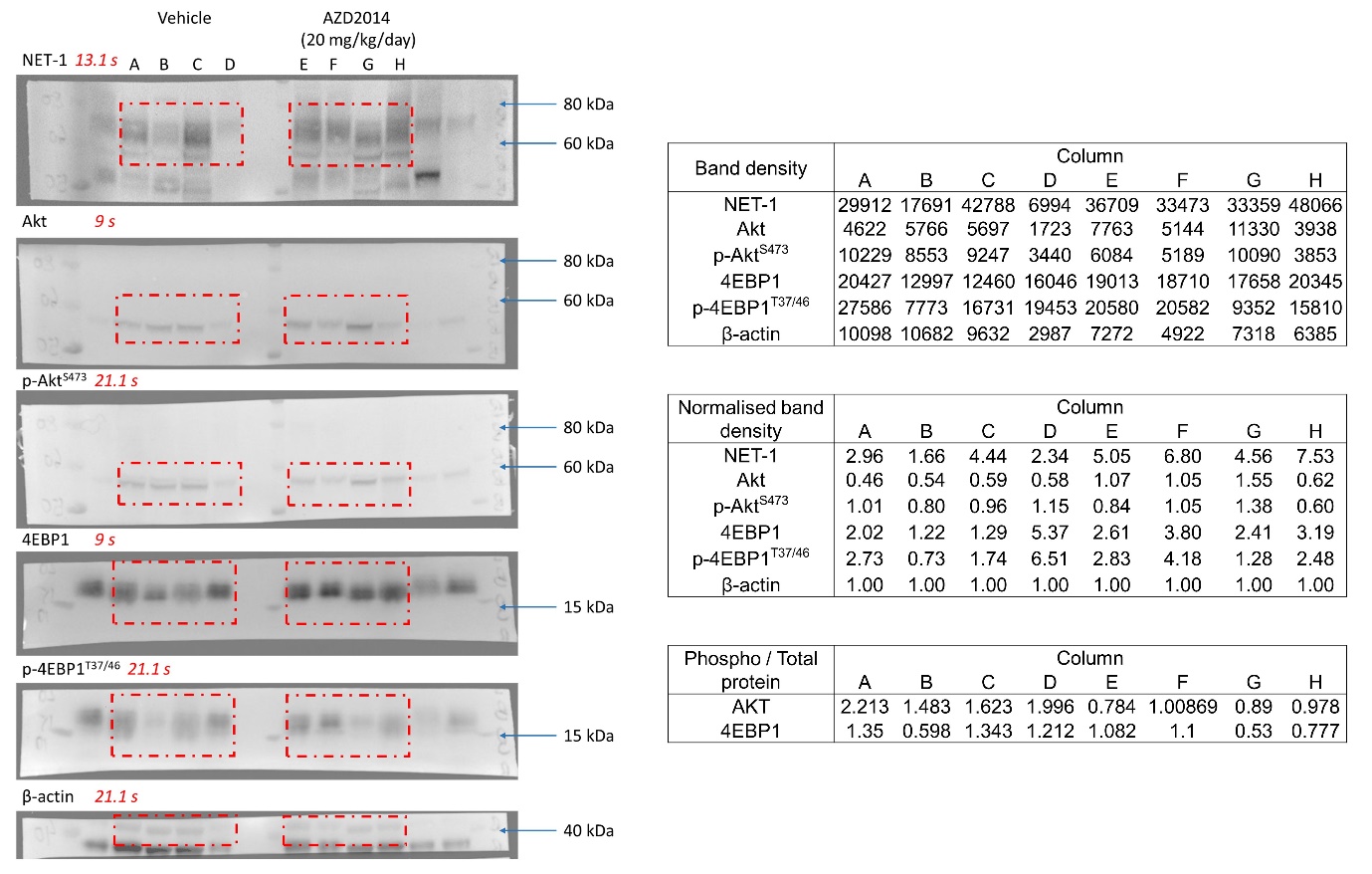


**WB3:** Immunoblots of SK-N-BE(2)C *in vivo* treatment with membrane photo overlay and band density for Figure 5b (Vehicle treatment = lanes A–D; AZD2014 treatment (20 mg/kg/day = lanes E–H). Red dashed boxes indicate the area used for the WB presented. Exposure time is indicated in red italics. Actual band density, intensity ratio (normalised to β-actin) and phosphorylated/total protein ratios are included in the tables to the right.

**For Figure 5d**


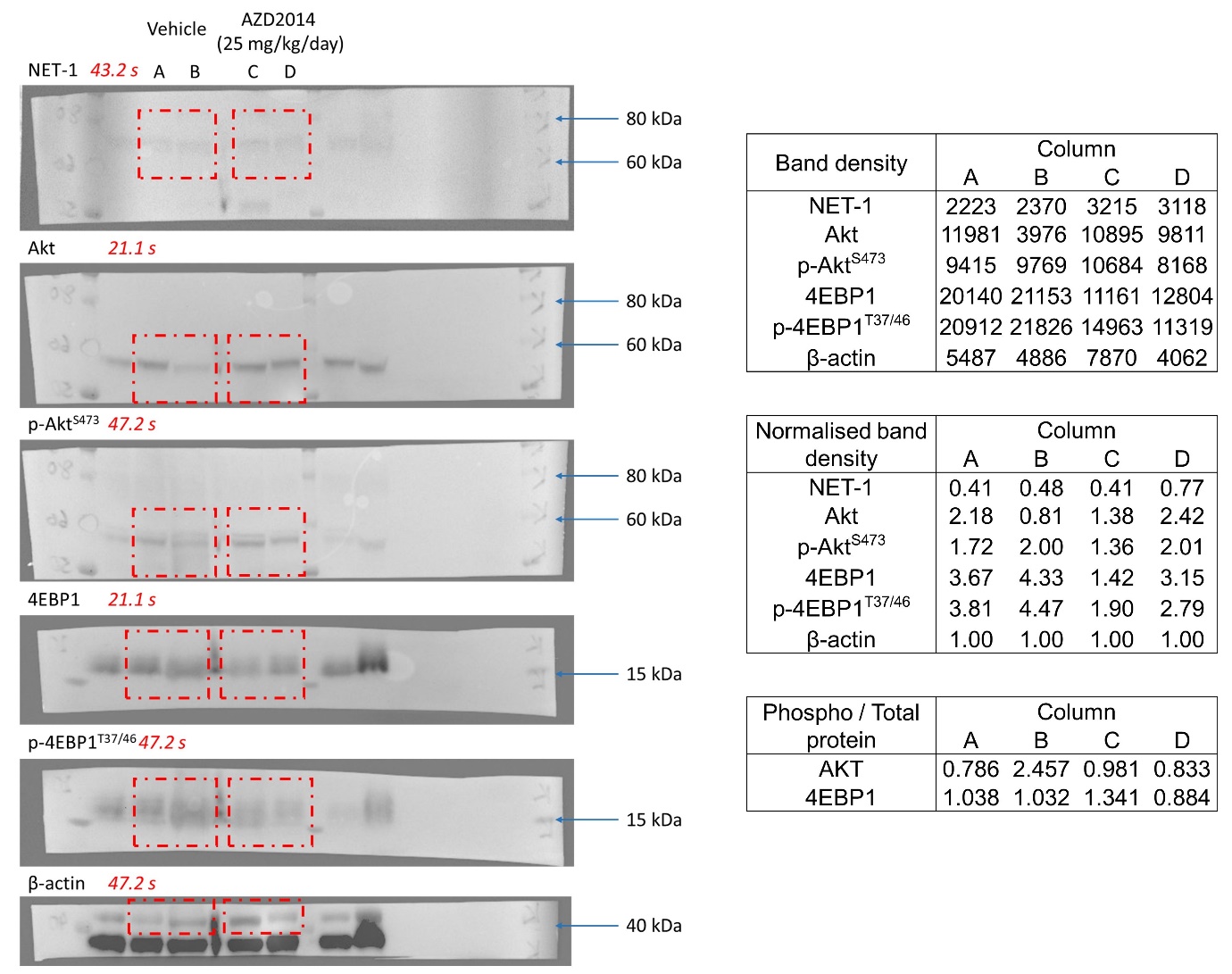


**WB4:** Immunoblots of Kelly *in vivo* treatment with membrane photo overlay and band density for Figure 5d (Vehicle treatment = lanes A/B; AZD2014 treatment (25 mg/kg/day = lanes C/D). Red dashed boxes indicate the area used for the WB presented. Exposure time is indicated in red italics. Actual band density, intensity ratio (normalised to β-actin) and phosphorylated/total protein ratios are included in the tables to the right.

**For Figure 4 and Supplementary Figure S6**


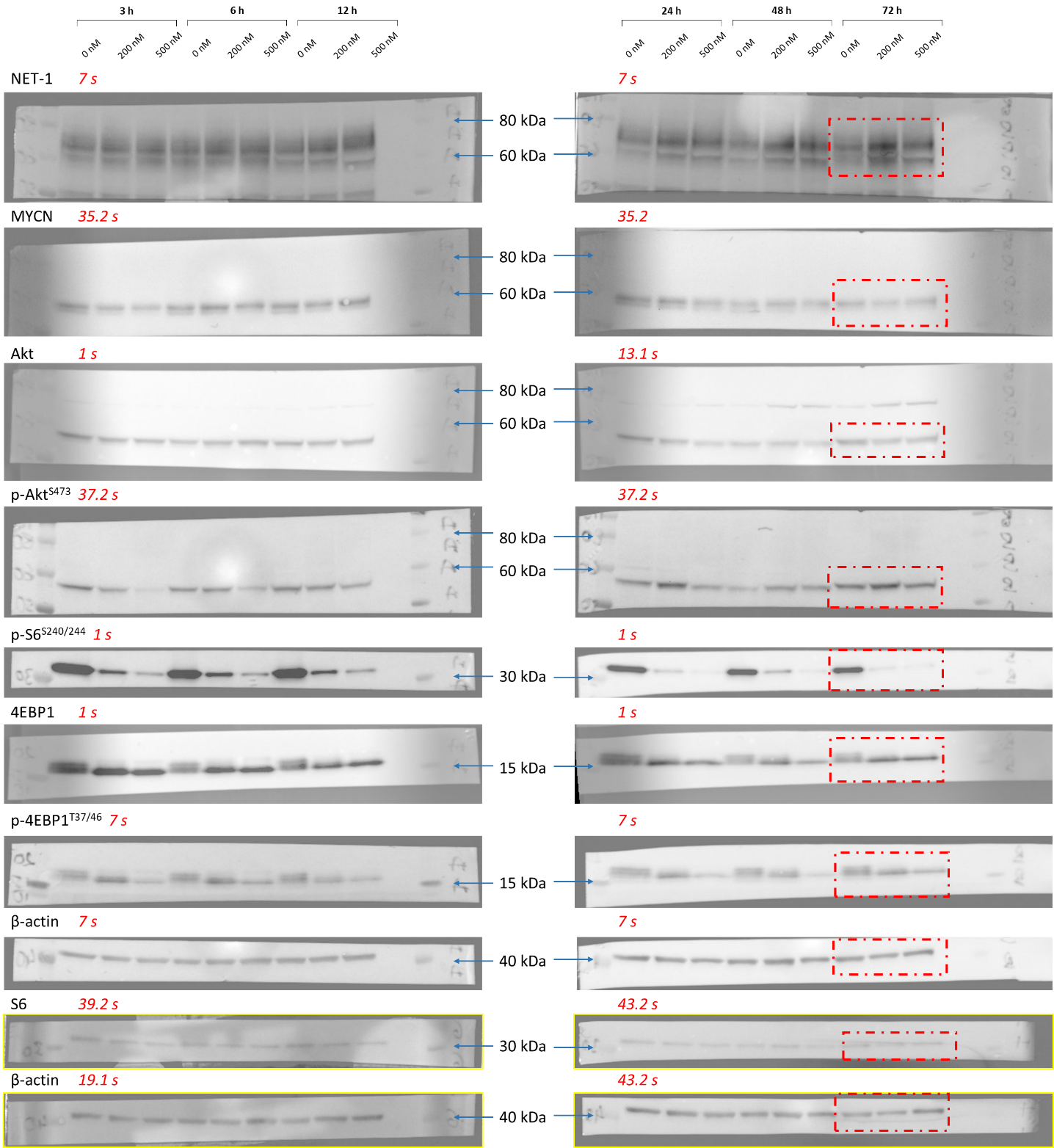


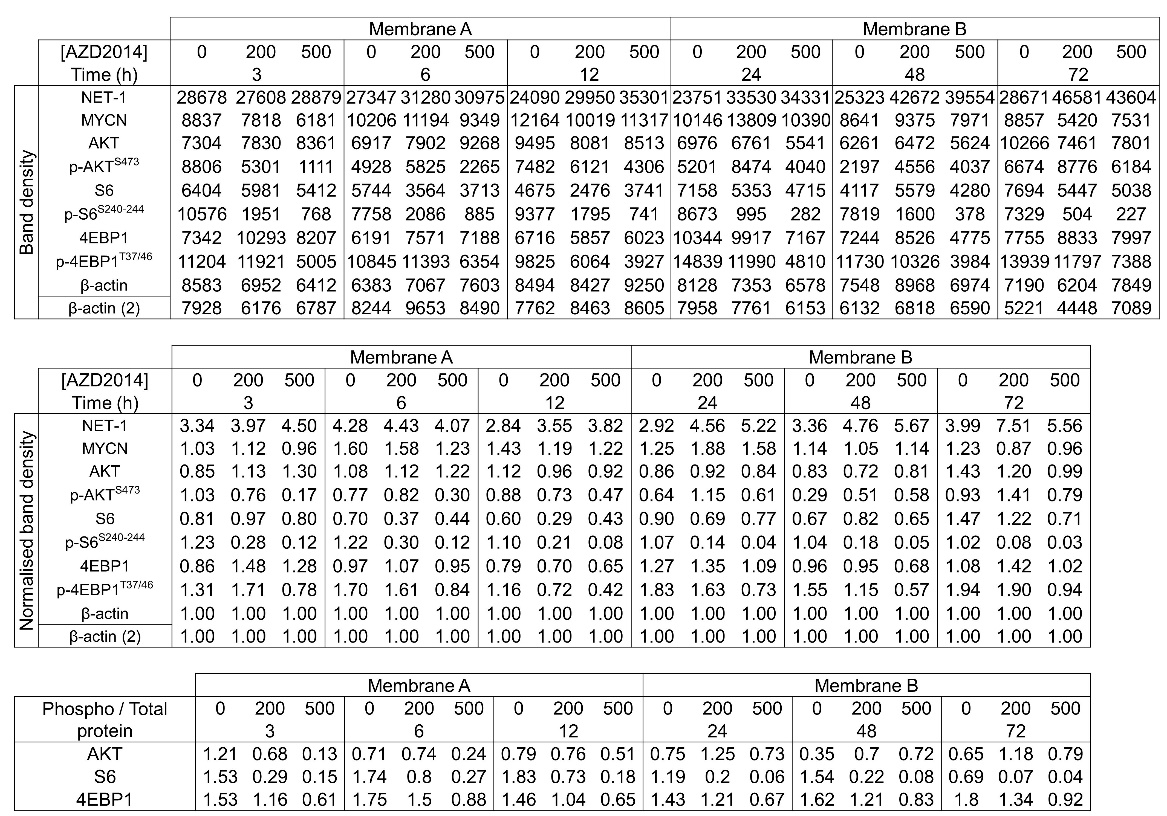


**WB5:** Immunoblots of SK-N-BE(2)C *in vitro* treatment with membrane photo overlay and band density for Figure 4 (red boxes) and supplementary Figure S6 (whole blot). Exposure time is indicated in red italics. Yellow outline indicates a second membrane was used of the same lysates for better protein signal acquisitions and a control band is included for this. Actual band density, intensity ratio (normalised to β-actin) and phosphorylated/total protein ratios are included in the tables.

**For Figure 4 and Supplementary Figure S7**


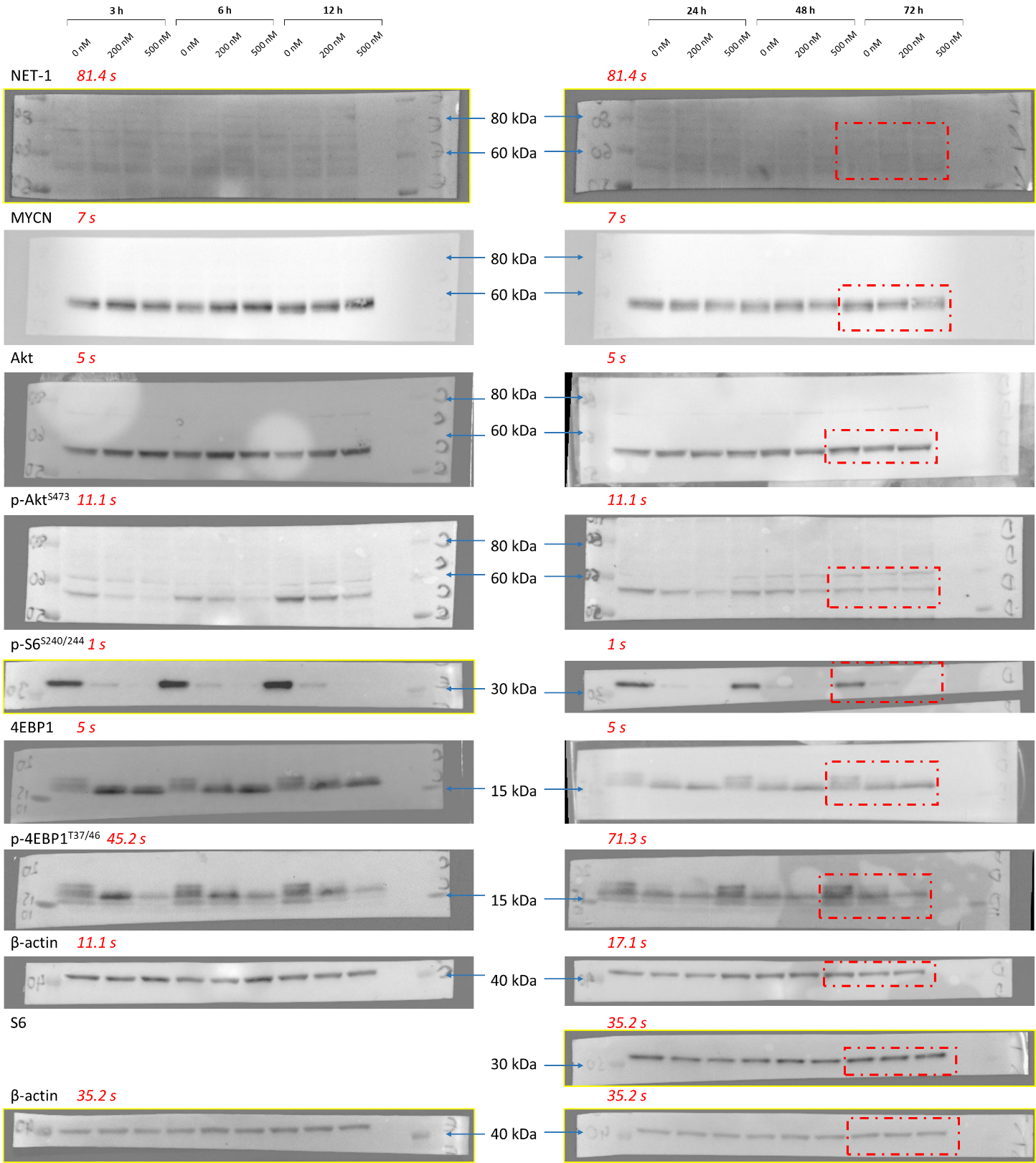


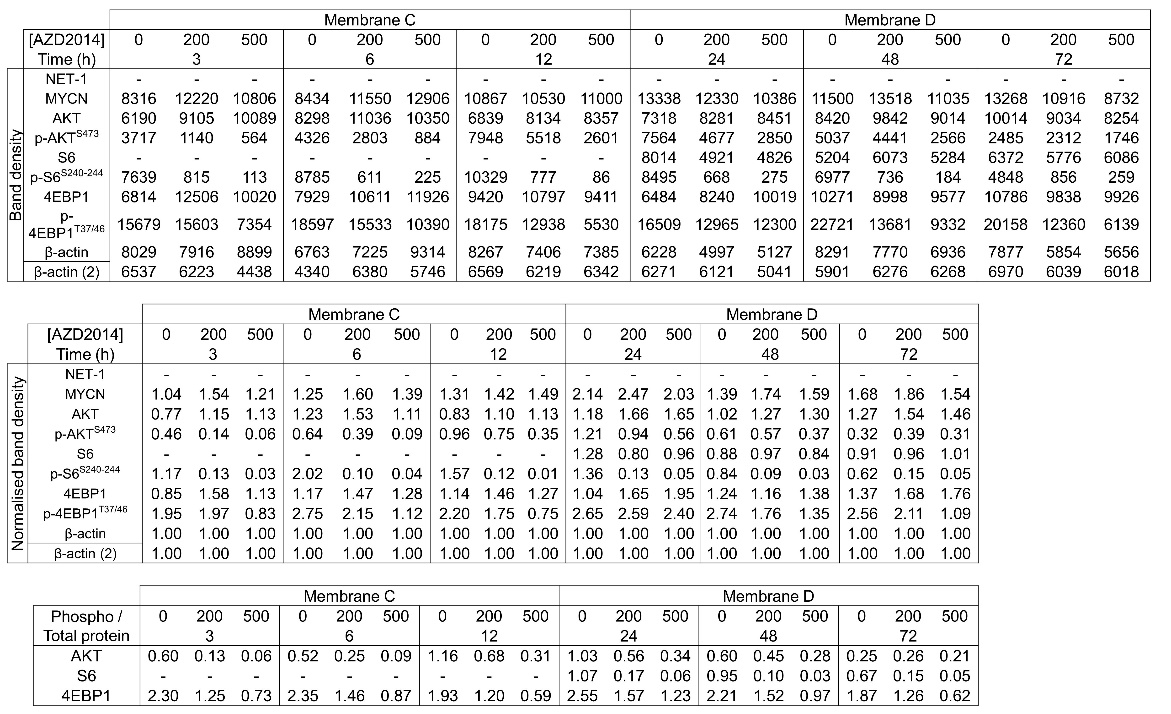


**WB6:** Immunoblots of Kelly *in vitro* treatment with membrane photo overlay and band density for Figure 4 (red boxes) and supplementary Figure S7 (whole blot). Exposure time is indicated in red italics. Yellow outline indicates a second membrane of the same lysates for better protein signal acquisitions and a control band is included for this. Actual band density, intensity ratio (normalised to β-actin) and phosphorylated/total protein ratios are included in the tables. No S6 band was captured the first blot (Membrane C – AZD2014 treatment 3–12 h in Kelly cells) owing to an artefact of stripping after p-s6 staining, therefore intensity ratios could not be performed here. NET-1 staining (Membranes C and D) was too diffuse and the signal too low to allow for accurate band intensity quantification.
